# Ultrasound-Assisted Synthesis of Fluorescent Azatetracyclic Derivatives: An Energy-Efficient Approach

**DOI:** 10.3390/molecules27103180

**Published:** 2022-05-16

**Authors:** Gheorghita Zbancioc, Catalina-Ionica Ciobanu, Ionel I. Mangalagiu, Costel Moldoveanu

**Affiliations:** 1 Chemistry Department, Alexandru Ioan Cuza University of Iasi, Carol I Bvd., 700506 Iasi, Romania; ionelm@uaic.ro; 2 Institute of Interdisciplinary Research-CERNESIM Centre, Alexandru Ioan Cuza University of Iasi, Carol I Bvd., 700506 Iasi, Romania; catalina.ciobanu@uaic.ro

**Keywords:** eco-friendly synthesis, ultrasound irradiation, heterogeneous catalytic bromination, azatetracyclic, azides, fluorescence

## Abstract

We report here an energy-efficient and straight synthesis of two new classes of derivatized fluorescent azatetracycles under ultrasound (US) irradiation. A first class of azatetracyclic compounds was synthesized by heterogeneous catalytic bromination of the α-keto substituent attached to the pyrrole moiety of the tetracyclic cycloadducts, while for the second, one class was synthesized by nucleophilic substitution of the bromide with the azide group. Comparative with conventional thermal heating (TH) under US irradiation, both types of reactions occur with substantially higher yields, shortened reaction time (from days to hours), lesser energy consumed, easier workup of the reaction, and smaller amounts of solvent required (at least three to five-fold less compared to TH), which make these reactions to be considered as energy efficient. The derivatized azatetracycle are blue emitters with λmax of fluorescence around 430–445 nm. A certain influence of the azatetracycle substituents concerning absorption and fluorescent properties was observed. Compounds anchored with a bulky azide group have shown decreased fluorescence intensity compared with corresponding bromides.

## 1. Introduction

In the recent years, ultrasound (US)-assisted reactions have become a modern and successful tool in organic and medicinal chemistry. US irradiation of organic reactions has several important advantages in terms of higher yields, increased purity of the compounds and selectivity, shorter reaction times, lower costs, and simplicity in handling and processing in comparison with conventional thermal heating (TH), being a facile alternative to several organic syntheses [1,2,3,4,5,6,7,8]. In addition, due to milder reaction conditions, less or no side reactions, the use of small amounts of solvents, and the energy-saving nature, the reaction can be considered eco-friendly [9,10,11]. 

Comparative with other derivatives, organic fluorophores have some undeniable advantages such us a wide spectral range, small size, great photostability, and, in many cases, high brightness. They are now widely used in drug discovery, cellular imaging, environmental analysis, and various medical applications. Probe structure can be varied to control excitation and emission wavelengths, target-binding affinity, chemical reactivity, and subcellular localization [12].

The structural arrangement of the azatetracycles, 18 π-electron N-fused heterocycles, containing a bridgehead nitrogen atom shared by an electron-excessive pyrrole and an azine electron-deficient six-membered ring, make them a pure blue-emitting moiety [13,14,15,16,17]. This uneven π-electron distribution between the two fused rings and the planar geometry (due to the sp^2^ hybridization of all the atoms in the fused ring) of the molecule is an important feature that leads to electron delocalization.

Moreover, the potential applications for medicinal chemistry as antimicrobial [18], antifungal [19], anticancer [20], and antituberculosis [21] agents and their fluorescence emission [22,23,24] increased the interest in the azaheterocycles field. The combined use of the two distinct properties (biological and optical) has suggested interesting applications as fluorescent biomarkers [13,25,26,27].

Our group focused on the dipolar cycloaddition reactions of azinium ylides with various dipolarophiles in order to obtain new compounds with fluorescent properties and biological activities [28,29,30,31,32,33,34,35,36]. We also added additional contributions in the field of using US irradiation in organic synthesis [1,2,3,4,5,37]. Herein, we propose a facile, efficient, and environmentally friendly method using US irradiation for the derivatization of previously obtained blue-emitting derivatives. The goal of the derivatization of these compounds was to increase their reactivity in order to make them easier to bind to various biomacromolecules of interest. Through this binding, the molecules can be labeled, facilitating their identification in order to study their mechanisms of action in the biological environment. Thus, these derivatives can be used as fluorescent azatetracyclic building blocks.

## 2. Results and Discussion

In previous studies [13], we halogenated the carbonyl group in α-position, obtaining halogenated derivatives with enhanced reactivity, which represent building blocks in the synthesis of fluorescent compounds. These derivatives can be incorporated into biological macromolecules (peptides, proteins, polysaccharides, lipides, and DNA) in order to fluorescent labeling those.

**Scheme 1:**
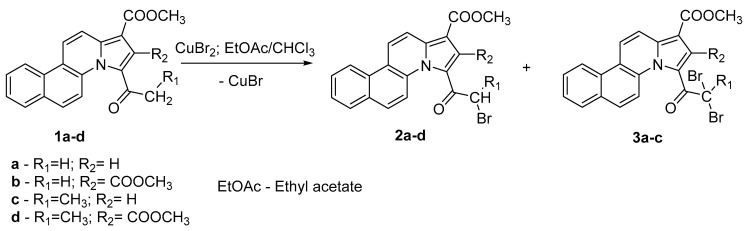
The α-bromination reaction of tetracyclic cycloadducts, **1a**–**d**.

In the present study, we applied this functionalization to the azatetracyclic derivatives that we previously obtained [37]. Thus, the tetracyclic cycloadduct products **1a**–**d** were subjected to the bromination in heterogeneous catalysis using copper (II) bromide in chloroform/ethyl acetate, as is presented in Scheme 1.

This reaction system is highly regioselective, leading only to α-bromo-derivatives, but less selective regarding to the resulting mono or dibrominated product. Using this procedure, we performed the bromination of cycloadducts **1a**–**d** with copper (II) bromide, both under conventional TH, and US irradiation in order to study the reaction selectivity. In Table 1, the optimized conditions we employed are listed.

As can be observed from Scheme 1 and Table 1, the major disadvantages of the bromination reaction under conventional TH are long reaction time (one day) and moderate yield (46 to 54%). In addition, under conventional TH, in the case of the **1a**–**c** tetracyclic derivatives bromination, both monobrominated **2a**–**c** (as major product) and dibrominated **3a**–**c** (as minor product) products were obtained. In the case of the **1d** tetracyclic derivative bromination, only the monobrominated **2d** product was obtained, most probably because of the steric hindrance between the carbomethoxy from the second position and the methyl from the propanoyl group.

Under US irradiation, these disadvantages were eliminated since the reaction time was shortened from one day to one hour, the yield was increased by 15 to 20%, and the most important, selectivity to the monobrominated product is increased to specificity (no dibrominated product was observed). In addition, the solvent amounts used under this procedure were five times lower than the corresponding quantities used under conventional conditions (see Experimental). Since the used solvent was a toxic halogenated compound, the reduction of its used amount qualifies the US-assisted reactions as less polluting.

The efficiency of US irradiation in the bromination reactions could be explained by the cavitation phenomena leading to enhanced mass transfer and better homogenization of the reaction mixture as previously reported [3].

**Scheme 2:**
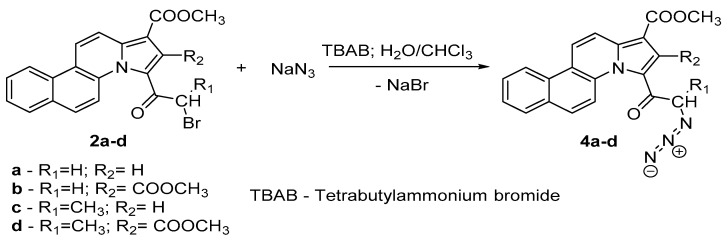
Synthesis reaction of tetracyclic azide derivatives **4a**–**d**.

The bromo derivatives with increased reactivity are valuable intermediary in synthetic chemistry, because they could be used as alkylating agents for direct chemical labelling of proteins targets cysteine and amine groups. Moreover, these brominated products can be easier converted to the corresponding azides using the procedure described in the literature [37,38]. The resulted azides are also highly valuable intermediary in synthetic chemistry since they can be used in the click chemistry, being extraordinary stabled toward H_2_O, O_2_, and the majority of organic synthesis condition [39]. In Scheme 2 is presented the reaction pathway for the synthesis of the new azides.

The reaction of the bromoderivatives with sodium azide in CHCl_3_/H_2_O mixture in the presence of the phase transfer catalyst tetrabutylammonium bromide (TBAB) took place at room temperature under vigorous stirring. The corresponding azides were obtained with good yield, but the major disadvantage was the reaction time (48 h).

To reduce the reaction time, we performed the reaction under US. In Table 2, the optimized condition and the corresponding yield are presented.

The data from Table 2 show that under US irradiation the reaction times decrease substantially from 2 days to 2 hours and the yield was improved by 10–15%. The efficiency of US irradiation in these reactions could be explained by the same cavitation phenomena. The solvent amounts used in the case of the US assisted reaction were at least three times lower than the corresponding quantities used under conventional conditions (see Experimental Section). Since the used solvent was a toxic halogenated compound, the reduction of its used amount qualifies the former reactions as less polluting.

The structures of compounds were proved through elemental and spectral analysis (FT-IR, ^1^H-NMR, ^13^C-NMR, and two-dimensional experiments 2D-COSY, HMQC, HMBC). The main argument that the substitution reaction of the bromine with azide group took place was found in the IR spectrum where the azide group present an absorption band in the range of 2200 cm^−1^, characteristic for this moiety (see Figures S19–S22 from Supplementary Materials). An additional proof for the azide formation is provided by the long range ^1^H-^15^N correlation HMBC spectrum, (see Figures S8d, S9d, S10d and S11d from Supplementary Materials) which shows that nitrogen atoms from the azide group is bonded to the methylene from α-position of the carbonyl group. The remaining signals in the FT-IR, ^1^H-NMR, and ^13^C-NMR spectra are all in accordance with the proposed structures.

Optical properties of the functionalized azatetracycles (bromides **2a**–**d** and azides **4a**–**d**) were investigated on diluted solutions (less than 10^−5^ mol/L) prepared in chloroform. The dilution of each solution was adjusted thus the absorbances maxima measured in the 340–440 nm range, and reported on a 10 mm cuvette, to fit in 0.5–0.6 unit range.

**Figure 1:**
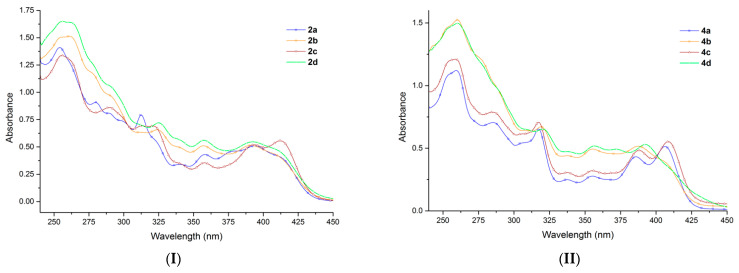
The absorption spectra recorded in chloroform solution of the functionalized derivatives: (**I**) bromides **2a**–**d** and (**II**) azides **4a**–**d**.

Since the compounds **2a**–**d** and **4a**–**d** have a relatively similar structure, they exhibit small differences in their experimental UV-Vis absorption spectra, as can be seen from Figure 1. The absorption maxima of the eight functionalized azatetracycles in chloroform, are summarized in Table 3. As we can observe in Table 3 and Figure 1, the position of the absorption bands depends on the substituents of the pyrrole ring. Thus, in the case of all compounds (Figure 1), three relatively well-separated absorption regions are observed (first between 240–300 nm, second between 300–335 nm and the third between 335–440 nm, respectively). The absorption bands from the first region have a higher (double or more) intensity than the absorption bands from the second region, and the latter ones have a higher (less than double) intensity than the absorption bands from the third region. The absorption bands responsible for the blue emission of the azatetracycles are situated in the third region (335–440 nm). In this region four more or less separated absorption bands are visible. A detailed discussion of the absorption spectra is presented in the Supplementary Materials.

**Figure 2:**
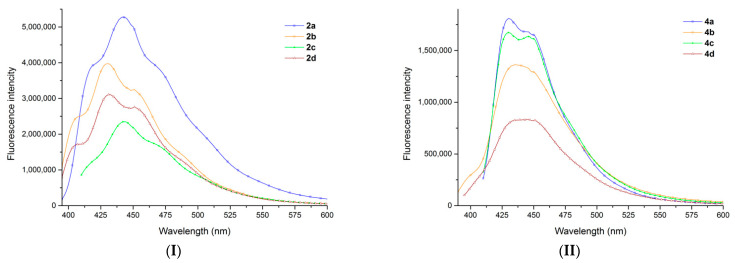
The emission spectra recorded in chloroform solution of the functionalized derivatives: bromides **2a**–**d** (**I**) and azides **4a**–**d** (**II**). Excitation wavelength was: 380 nm for sample **4b**; 385 nm for samples **2a**, **2b**, **2d**, and **4d**; and 400 nm for samples **2c**, **4a**, and **4c**.

All the studied tetracyclic derivatives have emission spectra consisting of one band situated in the 400–510 nm region (Figure 2). For the samples **2a**–**d**, **4a**, and **4c** the emission band is structured indicating a planar structure of the molecules, while in the case of the samples **4b** and **4d** this band is unstructured. A more detailed discussion on the absorption vs. emission spectra of the eight derivatives is presented in the Supplementary Matterials (see Figure S27).

The position of the band is significantly influenced by the presence of a carbomethoxy group at the 2nd position of the azatetracyclic skeleton. For the obtained bromides **2a**–**d**, a hypsochromic shift of Δ_max_ = 12 nm (**2a** compared with **2b**) and Δ_max_ = 11 nm (**2c** compared with **2d**) could be observed in emission spectra, while for the obtained azides **4a**–**d**, a bathochromic shift of Δ_max_ = 5 nm (**4a** compared with **4b**) and Δ_max_ = 15 nm (**4c** compared with **4d**) could be observed (Table 3 and Figure 2). 

This different behavior of the bromides vs. azides, in terms of: the form of the absorption band (structured versus unstructured), fluorescence intensity (higher versus lower), and the hypsochromic versus bathochromic shift in the emission spectra, can be explained by their structural difference, azide group being bulkier than the bromide. The bulky volume of the azide increases the steric hindrance between the carbomethoxy group at the second position of the azatetracyclic skeleton and the azide group, which determine part of the molecule to exit out of a plane. The alteration of the planar structure of the molecule determines a decrease in fluorescence. This fact can be observed when comparing the fluorescence intensity of the bromides **2a**–**d** versus azides **4a**–**d**, and also if we compare the fluorescence intensity of the underivatized cycloadducts **1a**–**d** with the fluorescence intensity of the derivatized azatetracyclic bromides **2a**–**d** or azides **4a**–**d** [37].

We consider that after the removal of the bromide group (by alkylation) or azide group (by click reaction), the fluorescence intensity of the new compounds should increase according to our previous experimental findings [17]. The increased reactivity and a possible increase of the fluorescence after binding determine us to consider that these derivatives can be used as fluorescent markers in biomolecule labeling.

Figure 2 presents the emission spectra in the chloroform of the bromides **2a**–**d** (left column) and of the azides **4a**–**d** (right column).

The optical absorption and emission maxima of the azatetracyclic derivatives in chloroform are summarized in Table 3.

Table 3 and Figure 2 show that all the compounds are blue emitters (λ_max_ of fluorescence around 430–445 nm). Compound **2a** has higher intensity in the emission spectra. Different behaviors (in terms of the maximum of the emission) of the samples **2a**, **2c** compared with **2b**, **2d** and of the samples **4a**, **4c** compared with **4b**, **4d** shown in Table 3 should relate to the difference in the electronic structures of **2a**, **2c** and **4a**, **4c** related to **2b**, **2d** and **4b**, **4d**, respectively. The fluorescence quantum yields of azatetracyclic bromides are higher than the fluorescence quantum yields of azatetracyclic azides. As seen in Table 3, bromides **2a**–**d** have a moderate to low quantum yield (22–16%), while azides **4a**–**d** have low quantum yield values (9–5%).

The fluorescence quantum yields of these compounds show their ability to serve as long-term monitoring fluorescent probes. This aspect is particularly important due to the continuous high demand in photostable fluorophores, especially for in vivo microscopy, to obtain high-quality images and to allow prolonged monitoring of same cells over time [32,40].

## 3. Experimental Section 

### 3.1. General Procedure

All the reagents and solvents were purchased from commercial sources and used without further purification except bromoacetone which was synthesized by the reaction of acetone with bromine in acetic acid as the catalyst. Melting points were recorded on an Electrothermal MEL-TEMP (Barnstead International, Dubuque, IA, USA) apparatus in open capillary tubes and are uncorrected. Analytical thin-layer chromatography was performed with commercial silica gel plates 60 F254 (Merck, Darmstadt, Germany) and visualized with UV light. The NMR spectra were recorded on a (Bruker, Vienna, Austria) Avance III 500 MHz spectrometer operating at 500 for ^1^H and 125 MHz for ^13^C. The following abbreviations were used to designate chemical shift multiplicities: s = singlet, d = doublet, t = triplet, m = multiplet. Chemical shifts were reported in delta (δ) units, part per million (ppm) and coupling constants (J) in Hz. Infrared (IR) data were recorded as films on potassium bromide (KBr) pellets on a FT-IR Vertex-70 (Bruker Optik, Leipzig, Germania) spectrophotometer. Ultrasound-assisted reactions were carried out using Bandelin Ultrasound reactor (Sonopuls GM 3200, Berlin, Germany), with a nominal power of 200 W and a frequency of 20 kHz. The booster horn SH 213 G was fixed tightly to the ultrasonic converter. The titanium flat probe tip TT13 (diameter: 12.7 mm; length: 7 mm) was fixed tightly to the booster horn. The titanium probe tip was immersed in the used solvent. The used reactor allowed us to control the pulse sequence, as well as the amplitude (mean percent of the nominal power) and the irradiation time. All these parameters are expected to influence the reaction. The reactions were performed using 5 s pulse on/5 s pulse off and 60% of the instrument nominal power. UV-Vis spectra were recorded on a (Shimadzu, Kyoto, Japan) 1800 PC spectrophotometer in chloroform (spectroscopic grade) solution. The fluorescence measurements were made using a (Edinburgh Instruments, Livingstone, UK) F900 photoluminescence spectrometer, in the same solvent as for the UV-Vis spectra, with the excitation wavelength set to the absorption band maximum. For all spectral determinations the solutions were kept in 10 mm path length quartz cells. The fluorescence quantum yield was determined at room temperature with an (Edinburgh Instruments, Livingstone, UK), with an integrating sphere and the excitation wavelength corresponds to the maximum of the absorption band.

#### 3.1.1. General Procedure for Synthesis of Brominated Azatetracyclic Derivatives, **2a**–**d** and **3a**–**c** under Conventional TH Conditions and US Irradiation 

Next, 1 mmol of cycloadduct **1a**–**d** (0.317 g for cycloadduct **1a**; 0.375 g for cycloadduct **1b**; 0.331 g for cycloadduct **1c**; 0.389 g for cycloadduct **1d**) dissolved in 30 mL mixture of chloroform/ethyl acetate (1:1 ratio) was added dropwise under stirring and refluxing, in one hour, to a suspension of 2 mmol copper (II) bromide (0.448 g) in 20 mL mixture of chloroform/ethyl acetate (1:1 ratio). The stirring and refluxing were continued for 24 h. The hot solution was filtered, in order to remove the copper (I) bromide that formed. The solvent was evaporated by vacuum distillation. The crude product was purified by column chromatography on silica gel (eluted with dichloromethane).

Under US irradiation, 1 mmol of cycloadduct **1a**–**d** (0.317 g for cycloadduct **1a**; 0.375 g for cycloadduct **1b**; 0.331 g for cycloadduct **1c**; 0.389 g for cycloadduct **1d**), and 2 mmol of copper (II) bromide (0.448 g), in 10 mL solvent (chloroform/ethyl acetate in 1:1 ratio), were placed in the reaction vessel and exposed to irradiation (from 1 h; see Table 1). Once the irradiation cycle was completed, the reaction tube was removed from the reactor, and processed as indicated above for TH condition.

#### 3.1.2. General Procedure for Synthesis of Functionalized Azatetracyclic Derivatives, **4a**–**d** under Conventional TH Conditions and US Irradiation

To a solution of 0.5 mmol of brominated azatetracyclic derivatives **2a**–**d** (0.198 g for **2a**; 0.227 g for **2b**; 0.205 g for **2c**; 0.234 g for **2d**) in chloroform (15 mL) 0.55 mmol of sodium azide (0.036 g), water (7.5 mL), and 0.12 mmol of tetrabutylammonium bromide (0.04 g) were added. The reaction mixture was stirred vigorously at room temperature for 48 h. After the reaction was finished (TLC), the reaction mixture was washed with water (3 × 25 mL), dried over magnesium sulfate and evaporated under reduced pressure to give the crude product. The purification of the crude product was done by column chromatography on silica gel, eluted with dichloromethane.

Under US irradiation, the mixture of 0.5 mmol of brominated azatetracyclic derivatives **2a**–**d** (0.198 g for **2a**; 0.227 g for **2b**; 0.205 g for **2c**; 0.234 g for **2d**) in chloroform (5 mL), 0.55 mmol of sodium azide (0.036 g), water (2.5 mL), and 0.12 mmol of tetrabutylammonium bromide (0.04 g) was placed into the double walls water cooled reaction vessel and exposed to irradiation (from 2 h; see Table 2). Once the irradiation cycle was completed, the reaction tube was removed from the reactor, and processed as indicated above for TH condition.

The spectral characterization of the obtained compounds is presented in the Supplementary Materials.

## 4. Conclusions

In conclusion, we report herein an energy-efficient method for obtaining two new classes of derivatized blue-fluorescent azatetracycles, both under conventional thermal heating and under unconventional ultrasound irradiation.

The synthesis of the derivatized fluorescent azatetracycles were performed in two steps: bromination in heterogeneous catalysis and nucleophilic substitution. 

Regarding the bromination reactions, we used the heterogeneous catalysis with copper (II) bromide in chloroform/ethyl acetate of the previously obtained cycloadducts. The bromination reactions occur regioselectively both under conventional and unconventional heating, leading only to α-brominated products with increased reactivity. Comparative with conventional TH, the bromination reactions under US irradiation occur with increased selectivity in regard to the monobrominated compound.

The brominated cycloadducts were used as an intermediary for the synthesis of corresponding azides azatetracycles derivatives, by using a nucleophilic substitution of the bromide atom with sodium azide in a chloroform/water mixture and TBAB as phase transfer catalyst. The reactions occur at room temperature under vigorous stirring, with good yield, but in a long reaction time (24 h). The use of US irradiation reduces the reaction time (2 h) and improves the reaction yield.

The use of the US irradiation in the case of both derivatization reactions (bromination and nucleophilic substitution with azide) offers several advantages in terms of yield, easier workup of the reaction, a substantial decrease in the consumed solvent, and a substantial reduction in reaction time (from days to hours) and thus a consequent diminution in energy consumption. Taking into consideration these advantages, the proposed method should be considered energy efficient and less polluting. The efficiency of US irradiation reactions could be explained by the cavitation phenomena leading to enhanced mass transfer and better homogenization of the reaction mixture.

The absorption and emission maxima of the obtained azatetracyclic derivatives were studied, these compounds being blue emitters. The absorption and emission spectra are dependent on their structure, and compounds with similar structure having small differences in their spectra. The bulky volume of the azide increases the steric hinderance with the carbomethoxy group at the second position and determine part of the molecule to exit out of a plane. The alteration of the planar structure of the molecule determines a decrease of the fluorescence. This fact can be observed when comparing the fluorescence intensity and fluorescence quantum yields of the bromides versus azides. 

## Figures and Tables

**Table 1:** The synthesis of brominated azatetracyclic derivatives under US irradiation and conventional TH.

Compound	Ultrasounds		Conventional TH	
	Reaction Time, (h)	Yield, %	Reaction Time, (h)	Yield, %
**2a**	1	87	24	52
**2b**	1	74	24	46
**2c**	1	85	24	53
**2d**	1	70	24	54
**3a**	1	0	24	10
**3b**	1	0	24	15
**3c**	1	0	24	11

**Table 2:** The synthesis of corresponding azides under US irradiation and room temperature stirring.

Compound	Ultrasounds		Room Temperature Stirring	
	Reaction Time, (h)	Yield, %	Reaction Time, (h)	Yield, %
**4a**	2	93	48	82
**4b**	2	86	48	70
**4c**	2	95	48	83
**4d**	2	83	48	68

**Table 3:** λ_max_ (nm) of absorption spectra, λ_max_ (nm) of emission spectra and fluorescence quantum yield (%) of compounds **2a**–**d** and **4a**–**d**.

Compound	Fluorescence (λ_max_, nm)	Absorption (λ_max_, nm)	Quantum Yields (%)
**2a**	443	394	22.11
**2b**	431	391	18.64
**2c**	444	412	15.92
**2d**	433	393	17.09
**4a**	430	406	9.34
**4b**	435	387	7.17
**4c**	430	409	8.99
**4d**	445	393	5.30

λ_exc_ = 380 nm for sample **4b**; λ_exc_ = 385 nm for samples **2a**, **2b**, **2d** and **4d**; λ_exc_ = 400 nm for samples **2c**, **4a** and **4c**.

## Supplementary Materials

The following supporting information can be downloaded at: https://www.mdpi.com/article/10.3390/molecules27103180/s1. Spectral characterization, details of the NMR (^1^H NMR, ^13^C NMR, ^1^H-^15^N HMBC), IR, absorption and emission spectra of the synthesized compounds can be found in the Supplementary Materials.

## Sample Availability

Samples of the compounds are not available from the authors.
