# Supplementary material for: Ultrasound-Assisted Synthesis of Fluorescent Azatetracyclic Derivatives: An Energy-Efficient Approach
Source: Molecules. 2022 May 16;27(10):3180. doi: 10.3390/molecules27103180 (PMC9147717; doi:10.3390/molecules27103180)
Supplement: Supplementary file 1 [file molecules-27-03180-s001.zip › molecules-1677829-supplementary.pdf]

molecules

Supplemenatary Materials

Ultrasound-Assisted Synthesis of Fluorescent Azatetracyclic Derivatives: An Energy-Efficient Approach

Gheorghita Zbancioc [1,*], Catalina-Ionica Ciobanu [2], Ionel I. Mangalagiu [1,2] and Costel Moldoveanu [1,*]

[1] Chemistry Department, Alexandru Ioan Cuza University of Iasi, 11 Carol 1st Bvd, 700506 Iasi, Romania; ionelm@uaic.ro
[2] Institute of Interdisciplinary Research-CERNESIM Centre, Alexandru Ioan Cuza University of Iasi, 11 Carol I, 700506 Iasi, Romania; catalina.ciobanu@uaic.ro
* Correspondence: gheorghita.zbancioc@uaic.ro (G.Z.); costel.moldoveanu@uaic.ro (C.M.); Tel.: +40-232-201278 (C.M.)

Contents

1. Spectral characterization of the obtained compounds. 2
2. NMR spectra of the obtained compounds. 6
3. IR spectra of the obtained compounds. 35
4. UV spectra of the obtained compounds 41
5. Absorption vs emission spectra of the obtained compounds 44

1. Spectral characterization of the obtained compounds

The following abbreviations were used to designate chemical shift multiplicities: s = singlet, d = doublet, dd = doublet of doublet, t = triplet, m = multiplet. Chemical shifts were reported in delta (δ) units, part per million (ppm) and coupling constants (*J*) in Hz.

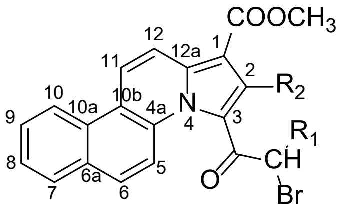

2a-d

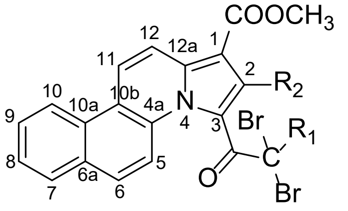

3a-c

a - R_1=H; R_2= H
b - R_1=H; R_2= $COOCH_3$
c - R_1=CH_3; R_2= H
d - R_1=CH_3; R_2= $COOCH_3$

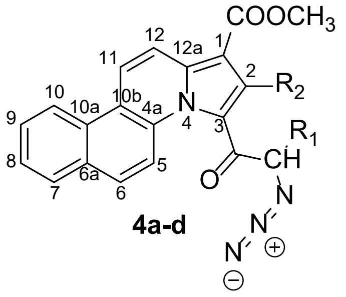

4a-d

a - R_1=H; R_2= H
b - R_1=H; R_2= $COOCH_3$
c - R_1=CH_3; R_2= H
d - R_1=CH_3; R_2= $COOCH_3$

Methyl 3-(2-bromoacetyl)benzo[f]pyrrolo[1,2-a]quinoline-1-carboxylate (2a). *(0.206 g, 52% (under conventional heating) and 0.345 g, 87% (under ultrasounds)) as a straw-yellow crystals, m.p. 194–195 °C); R_f (99.5/0.5 CH_2Cl_2/CH_3OH) 0.70;* ***IR*** *(cm^{-1}): 3067, 3032 (C-H arom.), 2952 (C-H aliph.), 1696 (C=O, ester), 1650 (C=O, keto), 1611, 1547, 1499, 1445, 1406 (aromatic and heteroaromatic ring), 1241, 1194, 1178, 1154, 1088, 1028 (C–O–C, ester), 642 (C–Br);* ***^{1}H NMR*** *(500 MHz, $CDCl_3$): δ 8.63 (1H, d, J = 9.5 Hz, H-11), 8.58 (1H, d, J = 8.0 Hz, H-10), 8.49 (1H, d, J = 9.5 Hz, H-12), 8.13 (1H, s, H-2), 8.01 (2H, m, overlapped peaks, H-5, H-6), 7.98 (1H, d, J = 8.0 Hz, H-7), 7.73 (1H, td, J = 7.0, 8.0 Hz, H-9), 7.65 (1H, t, J = 7.0, 8.0 Hz, H-8), 4.53 (2H, s, CH_2 of bromoacetyl group from 3rd position), 3.98 (3H, s, CH_3 of methoxycarbonyl group from 1st position);* ***^{13}C NMR*** *(125 MHz, $CDCl_3$): δ 180.6 (CO keto group from 3rd position), 164.2 (CO keto ester from 1st position), 141.2 (C-12a), 132.4 (C-4a), 131.1 (C-6a), 129.8 (C-6), 129.6 (C-10a), 129.0 (C-7), 128.6 (C-2), 127.9 (C-9), 127.0 (C-8), 125.8 (C-3), 125.1 (C-11), 123.0 (C-10), 121.4 (C-10b), 119.7 (C-5), 117.4 (C-12), 107.4 (C-1), 51.7 (CH_3 of methoxycarbonyl group from 1st position), 32.3 (CH_2 of bromoacetyl group from 3rd position). Anal. calc. for $C_{20}H_{14}BrNO_3$ (396.24): C 60.62, H 3.56, N 3.53; found: C 60.56, H 3.51, N 3.50.*

Dimethyl 3-(2-bromoacetyl)benzo[f]pyrrolo[1,2-a]quinoline-1,2-dicarboxylate (2b). *(0.209 g, 46% (under conventional heating) and 0.336 g, 74% (under ultrasounds)) as a yellow crystals, m.p. 174–175 °C); R_f (99.5/0.5 CH_2Cl_2/CH_3OH) 0.53;* ***IR*** *(cm^{-1}): 3104, 3024, 3000 (C-H arom.), 2949, 2928 (C-H aliph.), 1727, 1708 (C=O, ester), 1633 (C=O, keto), 1612, 1543, 1484, 1468, 1442, 1399, 1351, 1355 (aromatic and heteroaromatic ring), 1230, 1176, 1157, 1092 (C–O–C, ester), 629 (C–Br);* ***^{1}H NMR*** *(500 MHz, $CDCl_3$): δ 8.46 (2H, m, overlapped peaks, H-10, H-11), 8.31 (1H, d, J = 9.5 Hz, H-12), 7.96 (1H, d, J = 9.0 Hz, H-6), 7.92 (1H, d, J = 8.0 Hz, H-7), 7.69 (1H, td, J = 7.0, 8.0 Hz, H-9), 7.61 (2H, m, overlapped peaks, H-5, H-8), 4.37 (2H, s, CH_2 of bromoacetyl group from 3rd position), 4.06 (3H, s, CH_3 of methoxycarbonyl group from 1st position), 3.94 (3H, s, CH_3 of methoxycarbonyl group from 2nd position);* ***^{13}C NMR*** *(125 MHz, $CDCl_3$): δ 183.5 (CO keto group from 3rd position), 166.5 (CO keto ester from 1st position), 163.3 (CO keto ester from 2nd position), 138.2 (C-12a), 131.4 (C-4a), 130.9 (C-6a), 130.4 (C-6), 130.2 (C-10a), 129.6 (C-2), 128.9 (C-7), 128.2 (C-9), 127.2 (C-8), 124.3 (C-11), 123.8 (C-3), 122.9 (C-10), 121.5 (C-10b), 118.3 (C-5), 117.7 (C-12), 105.4 (C-1), 53.6 (CH_3 of methoxycarbonyl group from 1st position), 52.0 (CH_3 of methoxycarbonyl group from 2nd position), 33.6 (CH_2 of bromoacetyl group*

from 3rd position). Anal. calc. for $C_{22}H_{16}BrNO_5$ (454.28): C 58.17, H 3.55, N 3.08; found: C 58.12, H 3.50, N 3.04.

Methyl 3-(2-bromopropanoyl)benzo[f]pyrrolo[1,2-a]quinoline-1-carboxylate (2c). *(0.217 g, 53% (under conventional heating) and 0.349 g, 85% (under ultrasounds)) as a straw-yellow crystals, m.p. 201–202 °C); R_f (99.5/0.5 CH_2Cl_2/CH_3OH) 0.70;* ***IR*** *(cm^{-1}): 3062, 3053, 3000 (C-H arom.), 2949, 2937 (C-H aliph.), 1709 (C=O, ester), 1641 (C=O, keto), 1609, 1545, 1505, 1447, 1413, 1355 (aromatic and heteroaromatic ring), 1237, 1231, 1158, 1122, 1086 (C–O–C, ester) 609 (C–Br);* ***^{1}H NMR*** *(500 MHz, $CDCl_3$): δ 8.58 (2H, m, overlapped peaks, H-10, H-11), 8.46 (1H, d, J = 9.5 Hz, H-12), 8.08 (1H, s, H-2), 8.01 (1H, d, J = 9.5 Hz, H-6), 7.95 (2H, m, overlapped peaks, H-5, H-7), 7.71 (1H, t, J = 7.5 Hz, H-9), 7.63 (1H, t, J = 7.5 Hz, H-8), 5.48 (2H, q, J = 6.5 Hz, CHBr of bromopropanoyl group from 3rd position), 3.97 (3H, s, CH_3 of methoxycarbonyl group from 1st position), 2.01 (3H, d, J = 6.5 Hz, CH_3 of bromopropanoyl group from 3rd position);* ***^{13}C NMR*** *(125 MHz, $CDCl_3$): δ 183.0 (CO keto group from 3rd position), 164.3 (CO keto ester from 1st position), 141.2 (C-12a), 132.3 (C-4a), 131.0 (C-6a), 129.8 (C-6), 129.6 (C-10a), 128.9 (C-7), 127.9 (C-9), 127.1 (C-2), 126.9 (C-8), 125.9 (C-3), 124.8 (C-11), 123.0 (C-10), 121.3 (C-10b), 119.5 (C-5), 117.4 (C-12), 107.1 (C-1), 51.7 (CH_3 of methoxycarbonyl group from 1st position), 45.1 (CHBr of bromopropanoyl group from 3rd position), 20.7 (CH_3 of bromopropanoyl group from 3rd position). Anal. calc. for $C_{21}H_{16}BrNO_3$ (410.27): C 61.48, H 3.93, N 3.41; found: C 61.42, H 3.88, N 3.37.*

Dimethyl 3-(2-bromopropanoyl)benzo[f]pyrrolo[1,2-a]quinoline-1,2-dicarboxylate (2d). *(0.253 g, 54% (under conventional heating) and 0.328 g, 70% (under ultrasounds)) as a yellow crystals, m.p. 162–163 °C); R_f (99.5/0.5 CH_2Cl_2/CH_3OH) 0.53;* ***IR*** *(cm^{-1}): 3057, 3036, 3007 (C-H arom.), 2952, 2932 (C-H aliph.), 1741, 1708 (C=O, ester), 1659 (C=O, keto), 1604, 1541, 1498, 1470, 1400, 1341 (aromatic and heteroaromatic ring), 1260, 1213,1180, 1133, 1100 (C–O–C, ester), 657 (C–Br);* ***^{1}H NMR*** *(500 MHz, $CDCl_3$): δ 8.26 (1H, d, J = 8.5 Hz, H-10), 8.22 (1H, d, J = 10.0 Hz, H-11), 8.07 (1H, d, J = 10.0 Hz, H-12), 7.86 (1H, d, J = 9.5 Hz, H-6), 7.79 (1H, d, J = 7.5 Hz, H-7), 7.66 (1H, d, J = 9.5 Hz, H-5), 7.55 (1H, t, J = 7.0 Hz, H-9), 7.49 (1H, t, J = 7.0 Hz, H-8), 5.09 (1H, q, J = 7.0 Hz, CHBr of bromopropanoyl group from 3rd position), 4.05 (3H, s, CH_3 of methoxycarbonyl group from 1st position), 3.91 (3H, s, CH_3 of methoxycarbonyl group from 2nd position), 1.95 (3H, d, J = 7.0 Hz, CH_3 of bromopropanoyl group from 3rd position);* ***^{13}C NMR*** *(125 MHz, $CDCl_3$): δ 185.6 (CO keto group from 3rd position), 166.6 (CO keto ester from 1st position), 163.3 (CO keto ester from 2nd position), 138.1 (C-12a), 131.2 (C-4a), 130.6 (C-6a), 130.2 (C-6), 129.6 (C-10a), 129.3 (C-2), 128.6 (C-7), 127.9 (C-9), 126.9 (C-8), 123.9 (C-11), 123.6 (C-3), 122.6 (C-10), 121.1 (C-10b), 117.9 (C-5), 117.3 (C-12), 104.9 (C-1), 53.4 (CH_3 of methoxycarbonyl group from 1st position), 51.9 (CH_3 of methoxycarbonyl group from 2nd position), 46.8 (CHBr of bromopropanoyl group from 3rd position), 20.4 (CH_3 of bromopropanoyl group from 3rd position). Anal. calc. for $C_{23}H_{18}BrNO_5$ (468.30): C 58.99, H 3.87, N 2.99; found: C 58.93, H 3.83, N 2.94.*

Methyl 3-(2,2-dibromoacetyl)benzo[f]pyrrolo[1,2-a]quinoline-1-carboxylate (3a). *(0.048 g, 10% (under conventional heating) and 0 g, 0% (under ultrasounds)) as a yellow crystals, m.p. 170–171 °C); R_f (99.5/0.5 CH_2Cl_2/CH_3OH) 0.68;* ***IR*** *(cm^{-1}): 3113, 3022, 2996 (C-H arom.), 2943(C-H aliph.), 1693 (C=O, ester), 1652 (C=O, keto), 1543, 1504, 1467, 1450, 1334 (aromatic and heteroaromatic ring), 1284, 1256, 1233, 1188, 1163, 1115 (C–O–C, ester), 698, 648 (C–Br);* ***^{1}H NMR*** *(500 MHz, DMSO): δ 9.06 (1H, d, J = 9.5 Hz, H-11), 8.88 (1H, d, J = 8.5 Hz, H-10), 8.66 (1H, s, H-2), 8.46 (1H, d, J = 9.5 Hz, H-12), 8.27 (1H, d, J = 9.5 Hz, H-6), 8.14 (1H, d, J = 7.5 Hz, H-7), 8.09 (1H, s, CH of dibromoacetyl group from 3rd position), 7.82 (2H, m, overlapped peaks, H-9, H-5), 7.76 (1H, t, J = 7.5 Hz, H-8), 3.92 (3H, s, CH_3 of methoxycarbonyl group from 1st position);* ***^{13}C NMR*** *(125 MHz, DMSO): δ 176.3 (CO keto group from 3rd position), 163.3 (CO keto ester from 1st position), 141.1 (C-12a), 131.3 (C-4a), 130.5 (C-6a), 130.2 (C-6), 129.1 (C-10a), 128.8 (C-7), 128.6 (C-2), 128.3 (C-9), 127.4 (C-8), 126.3 (C-11), 123.6 (C-10), 121.4 (C-10b), 120.9 (C-3), 118.5 (C-5), 117.0 (C-12), 106.8 (C-1), 51.6 (CH_3 of methoxycarbonyl group from 1st position), 44.4 (CH of dibromoacetyl group from 3rd position). Anal. calc. for $C_{20}H_{13}Br_2NO_3$ (475.14): C 50.56, H 2.76, N 2.95; found: C 50.51, H 2.73, N 2.90.*

Dimethyl 3-(2,2-dibromoacetyl)benzo[f]pyrrolo[1,2-a]quinoline-1,2-dicarboxylate (3b). *(0.080 g, 15% (under conventional heating) and 0 g, 0% (under ultrasounds)) as a yellow-orange*

crystals, m.p. 178–179 °C); R_f (99.5/0.5 CH_2Cl_2/CH_3OH) 0.60; ***IR*** *(cm^{-1}): 3047, 3037, 3003, (C-H arom.), 2944, 2930 (C-H aliph.), 1733, 1717 (C=O, ester), 1669 (C=O, keto), 1610, 1542, 1468, 1441, 1401, 1356, 1338 (aromatic and heteroaromatic ring), 1251, 1219, 1184, 1123, 1097 (C–O–C, ester), 652, 639 (C–Br);* ***^{1}H NMR*** *(500 MHz, $CDCl_3$): δ 8.56 (2H, m, overlapped peaks, H-10, H-11), 8.39 (1H, d, J = 9.5 Hz, H-12), 8.04 (1H, d, J = 9.5 Hz, H-6), 7.97 (1H, d, J = 7.5 Hz, H-7), 7.73 (2H, m, overlapped peaks, H-9, H-5), 7.66 (1H, t, J = 7.5 Hz, H-8), 6.70 (1H, s, CH of dibromoacetyl group from 3rd position), 4.07 (3H, s, CH_3 of methoxycarbonyl group from 1st position), 3.96 (3H, s, CH_3 of methoxycarbonyl group from 2nd position);* ***^{13}C NMR*** *(125 MHz, $CDCl_3$): δ 178.5 (CO keto group from 3rd position), 166.2 (CO keto ester from 1st position), 163.1 (CO keto ester from 2nd position), 139.1 (C-12a), 131.6 (C-4a), 131.1 (C-6a), 131.0 (C-6), 131.0 (C-10a), 129.7 (C-2), 129.1 (C-7), 128.4 (C-9), 127.4 (C-8), 125.0 (C-11), 123.0 (C-10), 121.7 (C-3), 119.6 (C-10b), 117.8 (C-12), 117.7 (C-5), 105.9 (C-1), 53.8 (CH_3 of methoxycarbonyl group from 1st position), 52.2 (CH_3 of methoxycarbonyl group from 2nd position), 41.6 (CH of dibromoacetyl group from 3rd position). Anal. calc. for $C_{22}H_{15}Br_2NO_5$ (533.17): C 49.56, H 2.84, N 2.63; found: C 49.50, H 2.80, N 2.60.*

Methyl 3-(2,2-dibromopropanoyl)benzo[f]pyrrolo[1,2-a]quinoline-1-carboxylate (3c). *(0.054 g, 11% (under conventional heating) and 0 g, 0% (under ultrasounds)) as a yellow crystals, m.p. 175–176 °C); R_f (99.5/0.5 CH_2Cl_2/CH_3OH) 0.68;* ***IR*** *(cm^{-1}): 3108, 3023 (C-H arom.), 2960, 2928 (C-H aliph.), 1712 (C=O, ester), 1633 (C=O, keto), 1594, 1546, 1495, 1440, 1408 (aromatic and heteroaromatic ring), 1262, 1237, 1150, 1083, 1032 (C–O–C, ester), 667 (C–Br);* ***^{1}H NMR*** *(500 MHz, DMSO): δ 9.02 (1H, d, J = 9.5 Hz, H-11), 8.87 (1H, d, J = 8.0 Hz, H-10), 8.69 (1H, s, H-2), 8.45 (1H, d, J = 9.5 Hz, H-12), 8.17 (1H, d, J = 9.5 Hz, H-6), 8.14 (1H, d, J = 8.0 Hz, H-7), 7.83 (1H, t, J = 7.5 Hz, H-9), 7.75 (1H, t, J = 7.5 Hz, H-8), 7.69 (1H, d, J = 9.5 Hz, H-5) 3.93 (3H, s, CH_3 of methoxycarbonyl group from 1st position), 2.89 (3H, s, CH_3 of dibromopropanoyl group from 3rd position);* ***^{13}C NMR*** *(125 MHz, DMSO): δ 180.6 (CO keto group from 3rd position), 163.3 (CO keto ester from 1st position), 139.7 (C-12a), 131.4 (C-4a), 130.4 (C-6a), 130.0 (C-6), 129.2 (C-10a), 129.1 (C-2), 128.8 (C-7), 128.2 (C-9), 127.3 (C-8), 126.0 (C-11), 123.6 (C-10), 122.4 (C-3), 120.9 (C-10b), 119.6 (C-5), 116.7 (C-12), 106.1 (C-1), 58.6 (CBr_2 of dibromopropanoyl group from 3rd position), 51.7 (CH_3 of methoxycarbonyl group from 1st position), 37.4 (CH_3 of dibromopropanoyl group from 3rd position). Anal. calc. for $C_{21}H_{15}Br_2NO_3$ (489.16): C 51.56, H 3.09, N 2.86; found: C 51.50, H 3.05, N 2.82.*

Methyl 3-(2-azidoacetyl)benzo[f]pyrrolo[1,2-a]quinoline-1-carboxylate (4a). *(0.147 g, 82% (under conventional heating) and 0.167 g, 93% (under ultrasounds)) as a yellow crystals, m.p. 189–190 °C); R_f (99.5/0.5 CH_2Cl_2/CH_3OH) 0.65;* ***IR*** *(cm^{-1}): 3121, 3017 (C-H arom.), 2955, 2924 (C-H aliph.), 2144, 2111 (N_3 azide), 1711 (C=O, ester), 1658 (C=O, keto), 1608, 1557, 1544, 1500, 1450, 1403, 1367 (aromatic and heteroaromatic ring), 1233, 1181, 1129, 1089 (C–O–C, ester);* ***^{1}H NMR*** *(500 MHz, $CDCl_3$): δ 8.65 (1H, d, J = 9.5 Hz, H-11), 8.60 (1H, d, J = 8.5 Hz, H-10), 8.51 (1H, d, J = 9.5 Hz, H-12), 8.12 (1H, d, J = 9.0 Hz, H-6), 8.09 (1H, s, H-2), 8.01 (2H, m, overlapped peaks, H-5, H-7), 7.74 (1H, t, J = 8.0 Hz, H-9), 7.66 (1H, t, J = 8.0 Hz, H-8), 4.60 (2H, s, CH_2 of azidoacetyl group from 3rd position), 3.98 (3H, s, CH_3 of methoxycarbonyl group from 1st position);* ***^{13}C NMR*** *(125 MHz, $CDCl_3$): δ 182.2 (CO keto group from 3rd position), 164.2 (CO keto ester from 1st position), 141.1 (C-12a), 132.5 (C-4a), 131.1 (C-6a), 129.8 (C-6), 129.6 (C-10a), 129.0 (C-7), 128.6 (C-2), 128.0 (C-9), 127.1 (C-8), 125.6 (C-3), 125.2 (C-11), 123.0 (C-10), 121.5 (C-10b), 120.0 (C-5), 117.4 (C-12), 107.6 (C-1), 55.5 (CH_2 of azidoacetyl group from 3rd position), 51.7 (CH_3 of methoxycarbonyl group from 1st position). Anal. calc. for $C_{20}H_{14}N_4O_3$ (358.36): C 67.03, H 3.94, N 15.63; found: C 66.96, H 3.90, N 15.56.*

Dimethyl 3-(2-azidoacetyl)benzo[f]pyrrolo[1,2-a]quinoline-1,2-dicarboxylate (4b). *(0.146 g, 70% (under conventional heating) and 0.179 g, 86% (under ultrasounds)) as a yellow-orange crystals, m.p. 137–138 °C); R_f (99.5/0.5 CH_2Cl_2/CH_3OH) 0.45;* ***IR*** *(cm^{-1}): 3004 (C-H arom.), 2962, 2929 (C-H aliph.), 2115, 2102 (N_3 azide), 1717, 1702 (C=O, ester), 1659 (C=O, keto), 1611, 1547, 1471, 1446, 1404, 1368 (aromatic and heteroaromatic ring), 1262, 1246, 1163, 1132, 1101, 1031 (C–O–C, ester);* ***^{1}H NMR*** *(500 MHz, $CDCl_3$): δ 8.60 (2H, m, overlapped peaks, H-10, H-11), 8.47 (1H, d, J = 9.5 Hz, H-12), 8.05 (1H, d, J = 9.5 Hz, H-6), 8.00 (1H, d, J = 7.5 Hz, H-7), 7.78 (1H, t, J = 7.0 Hz, H-9), 7.69 (1H, t, J = 7.0 Hz, H-8), 7.63 (1H, d, J = 9.5 Hz, H-5), 4.33 (2H,*

s, CH_2 of azidoacetyl group from 3rd position), 4.05 (3H, s, CH_3 of methoxycarbonyl group from 1st position), 3.96 (3H, s, CH_3 of methoxycarbonyl group from 2nd position); ***13C NMR*** *(125 MHz, $CDCl_3$): δ 186.7 (CO keto group from 3rd position), 166.3 (CO keto ester from 1st position), 163.4 (CO keto ester from 2nd position), 138.1 (C-12a), 131.6 (C-4a), 131.0 (C-6a), 130.6 (C-6), 130.3 (C-10a), 129.8 (C-2), 129.1 (C-7), 128.4 (C-9), 127.4 (C-8), 124.3 (C-11), 124.0 (C-3), 123.1 (C-10), 121.7 (C-10b), 118.5 (C-5), 118.0 (C-12), 105.7 (C-1), 56.6 (CH_2 of azidoacetyl group from 3rd position), 53.6 (CH_3 of methoxycarbonyl group from 1st position), 52.1 (CH_3 of methoxycarbonyl group from 2nd position). Anal. calc. for $C_{22}H_{16}N_4O_5$ (416.39): C 63.46, H 3.87, N 13.46; found: C 63.40, H 3.84, N 13.40.*

Methyl 3-(2-azidopropanoyl)benzo[f]pyrrolo[1,2-a]quinoline-1-carboxylate (4c). *(0.155 g, 83% (under conventional heating) and 0.177 g, 95% (under ultrasounds)) as a yellow crystals, m.p. 167–168 °C); R_f (99.5/0.5 CH_2Cl_2/CH_3OH) 0.65;* ***IR*** *(cm^{-1}): 3078, 3025 (C-H arom.), 2974. 2949, 2921 (C-H aliph.), 2110, 2088, 2063 (N_3 azide), 1693 (C=O, ester), 1645 (C=O, keto), 1609, 1544, 1507, 1494, 1439, 1414, 1404, 1322 (aromatic and heteroaromatic ring), 1238, 1176, 1085, 1051, 1031 (C–O–C, ester);* ***1H NMR*** *(500 MHz, $CDCl_3$): δ 8.44 (2H, m, overlapped peaks, H-10, H-11), 8.33 (1H, d, J = 9.5 Hz, H-12), 8.04 (1H, s, H-2), 7.92 (1H, d, J = 9.5 Hz, H-6), 7.89 (1H, d, J = 8.0 Hz, H-7), 7.84 (1H, d, J = 9.5 Hz, H-5), 7.63 (1H, t, J = 7.5 Hz, H-9), 7.57 (1H, t, J = 7.5 Hz, H-8), 4.71 (2H, q, J = 7.0 Hz, CHN_3 of azidopropanoyl group from 3rd position), 3.95 (3H, s, CH_3 of methoxycarbonyl group from 1st position), 1.73 (3H, d, J = 7.0 Hz, CH_3 of azidopropanoyl group from 3rd position);* ***13C NMR*** *(125 MHz, $CDCl_3$): δ 185.6 (CO keto group from 3rd position), 164.0 (CO keto ester from 1st position), 141.1 (C-12a), 132.1 (C-4a), 130.8 (C-6a), 129.6 (C-6), 129.4 (C-10a), 128.8 (C-7), 128.6 (C-9), 127.8 (C-2), 126.9 (C-8), 125.6 (C-3), 124.8 (C-11), 122.8 (C-10), 121.2 (C-10b), 119.6 (C-5), 117.2 (C-12), 107.4 (C-1), 59.6 (CHN_3 of azidopropanoyl group from 3rd position), 51.6 (CH_3 of methoxycarbonyl group from 1st position), 17.3 (CH_3 of azidopropanoyl group from 3rd position). Anal. calc. for $C_{21}H_{16}N_4O_3$ (372.38): C 67.73, H 4.33, N 15.05; found: C 67.67, H 4.29, N 14.99.*

Dimethyl 3-(2-azidopropanoyl)benzo[f]pyrrolo[1,2-a]quinoline-1,2-dicarboxylate (4d). *(0.146 g, 68% (under conventional heating) and 0.179 g, 83% (under ultrasounds)) as a yellow-orange crystals, m.p. 160–161 °C); R_f (99.5/0.5 CH_2Cl_2/CH_3OH) 0.45;* ***IR*** *(cm^{-1}): 3034, 3014 (C-H arom.), 2950, 2930 (C-H aliph.), 2131, 2114, 2100 (N_3 azide), 1732, 1697 (C=O, ester), 1665 (C=O, keto), 1610, 1544, 1503, 1468, 1442, 1408 (aromatic and heteroaromatic ring), 1264, 1228, 1189, 1159, 1094, 1074 (C–O–C, ester);* ***1H NMR*** *(500 MHz, $CDCl_3$): δ 8.57 (1H, d, J = 8.0 Hz, H-10), 8.53 (1H, d, J = 9.5 Hz, H-11), 8.43 (1H, d, J = 9.5 Hz, H-12), 8.04 (1H, d, J = 9.0 Hz, H-6), 7.98 (1H, d, J = 8.0 Hz, H-7), 7.75 (1H, t, J = 7.5, 8.0 Hz, H-9), 7.66 (1H, t, J = 7.5, 8.0 Hz, H-8), 7.58 (1H, d, J = 9.0 Hz, H-5), 4.30 (1H, q, J = 7.0 Hz, CHN_3 of azidopropanoyl group from 3rd position), 4.03 (3H, s, CH_3 of methoxycarbonyl group from 1st position), 3.95 (3H, s, CH_3 of methoxycarbonyl group from 2nd position), 1.56 (3H, d, J = 7.0 Hz, CH_3 of azidopropanoyl group from 3rd position);* ***13C NMR*** *(125 MHz, $CDCl_3$): δ 190.7 (CO keto group from 3rd position), 166.1 (CO keto ester from 1st position), 163.4 (CO keto ester from 2nd position), 137.7 (C-12a), 131.4 (C-4a), 130.9 (C-6a), 130.7 (C-6), 130.0 (C-10a), 129.7 (C-2), 129.1 (C-7), 128.4 (C-9), 127.3 (C-8), 123.9 (C-3), 123.8 (C-11), 123.0 (C-10), 121.5 (C-10b), 118.2 (C-5), 118.1 (C-12), 105.5 (C-1), 60.9 (CHN_3 of azidopropanoyl group from 3rd position), 53.4 (CH_3 of methoxycarbonyl group from 1st position), 52.1 (CH_3 of methoxycarbonyl group from 2nd position), 16.7 (CH_3 of azidopropanoyl group from 3rd position). Anal. calc. for $C_{23}H_{18}N_4O_5$ (430.42): C 64.18, H 4.22, N 13.02; found: C 64.13, H 4.18, N 12.97.*

2. NMR spectra of the obtained compounds

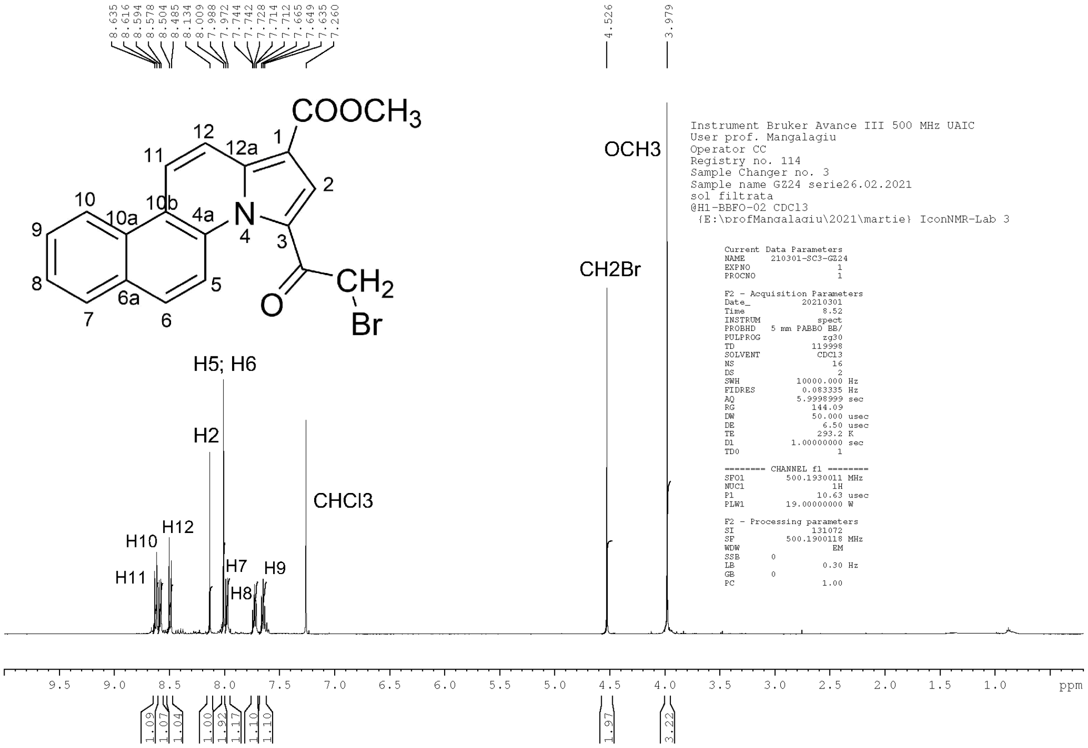

Figure S1a. ^{1}H NMR spectrum of the compound **2a.**

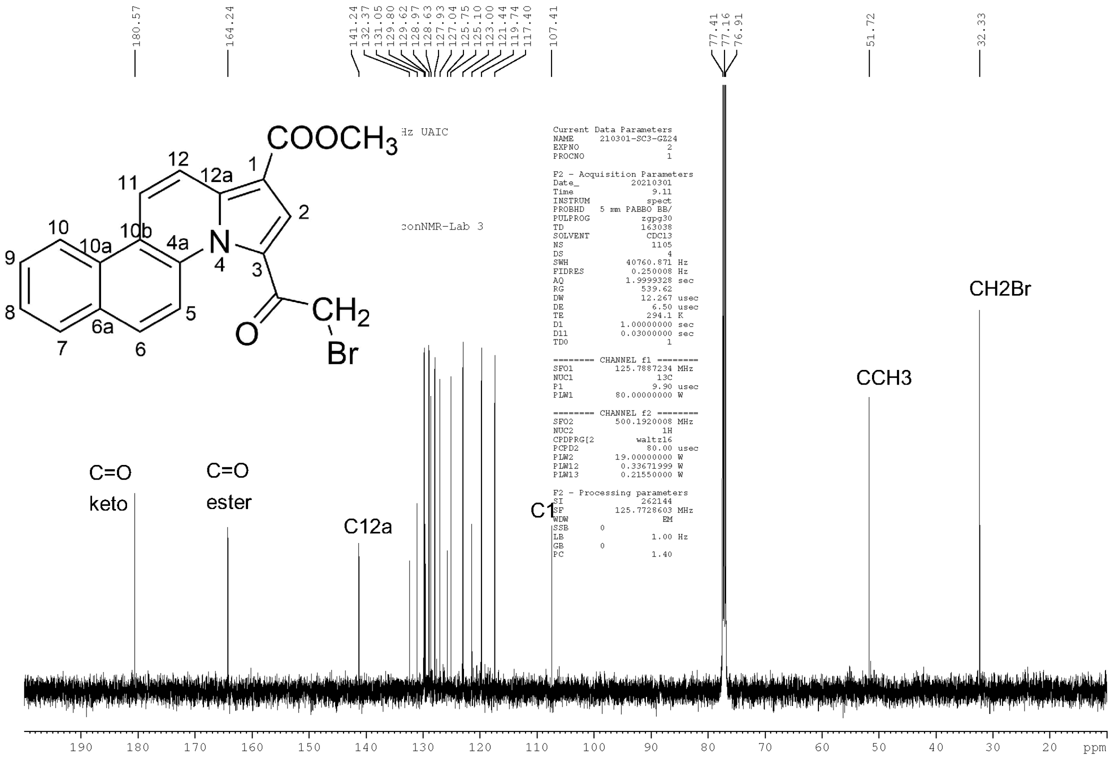

Figure S1b. ^{13}C NMR spectrum of the compound **2a.**

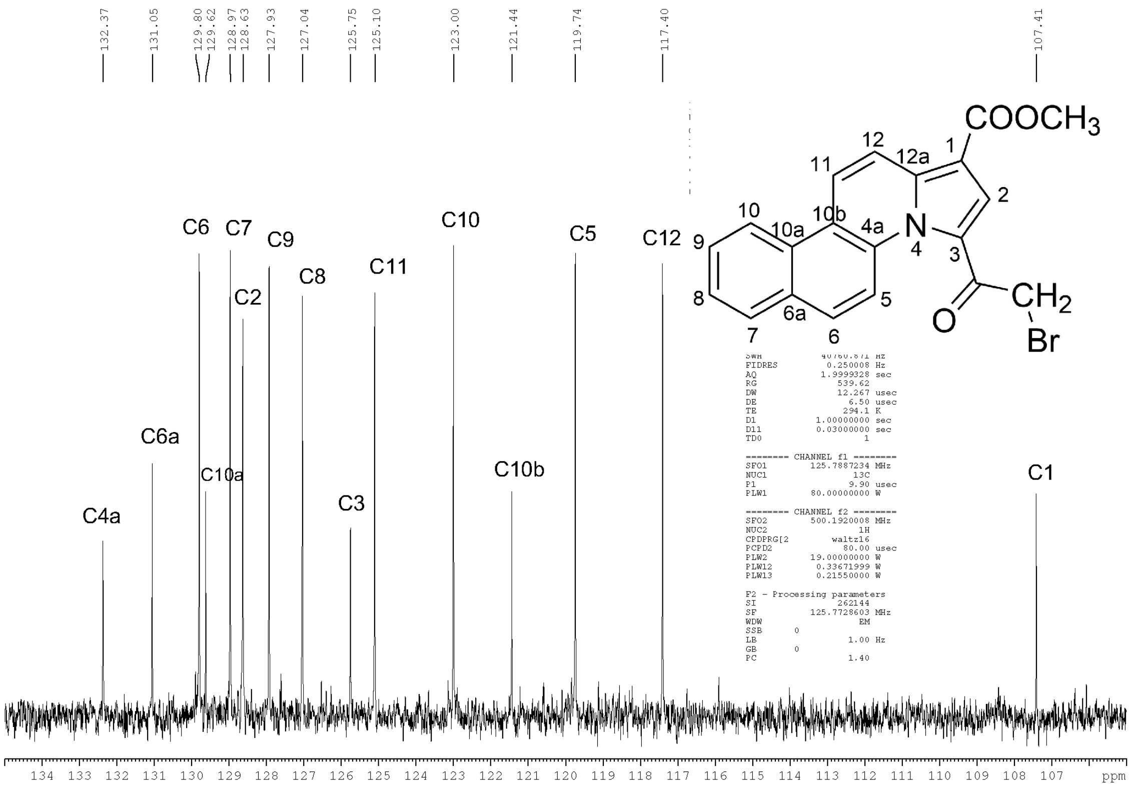

Figure S1c. Detail in the aromatic area of the ^{13}C NMR spectrum of the compound **2a.**

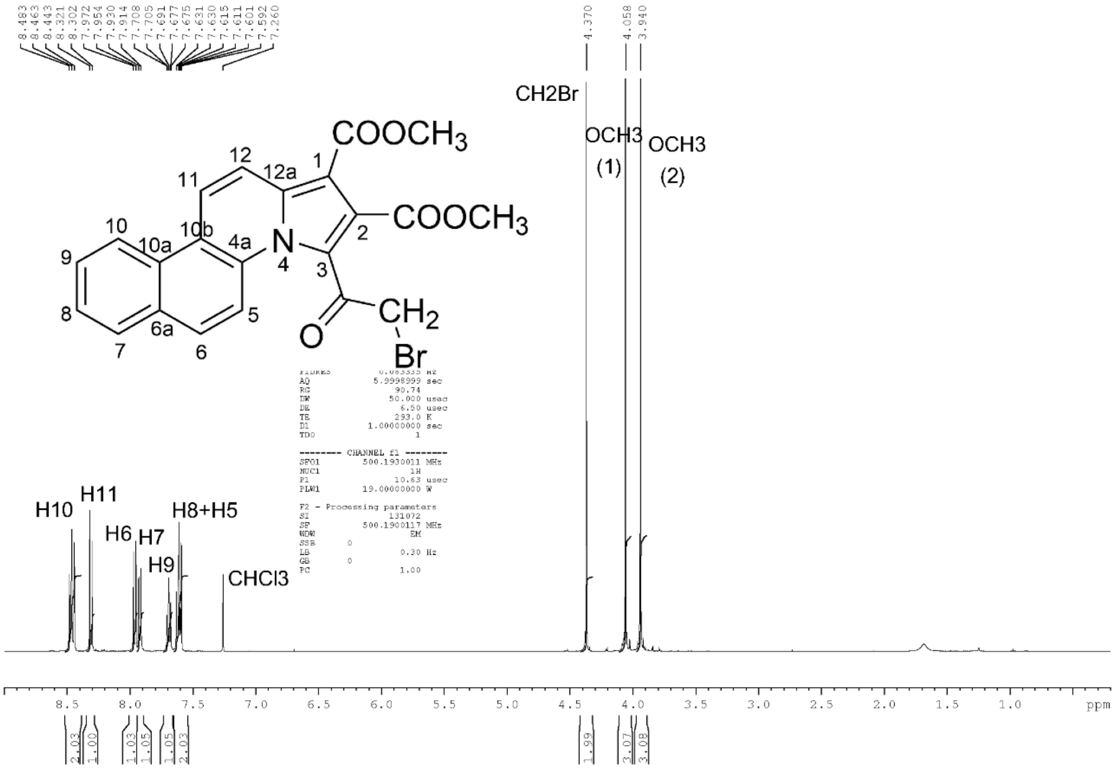

Figure S2a. ^{1}H NMR spectrum of the compound **2b.**

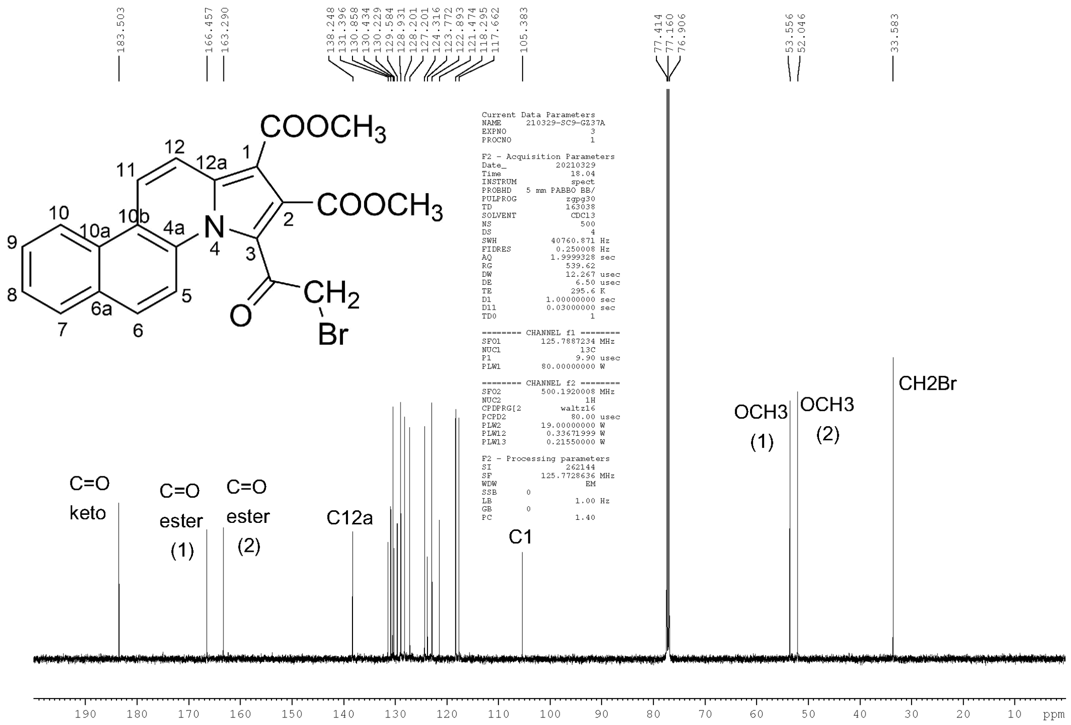

Figure S2b . ^{13}C NMR spectrum of the compound **2b.**

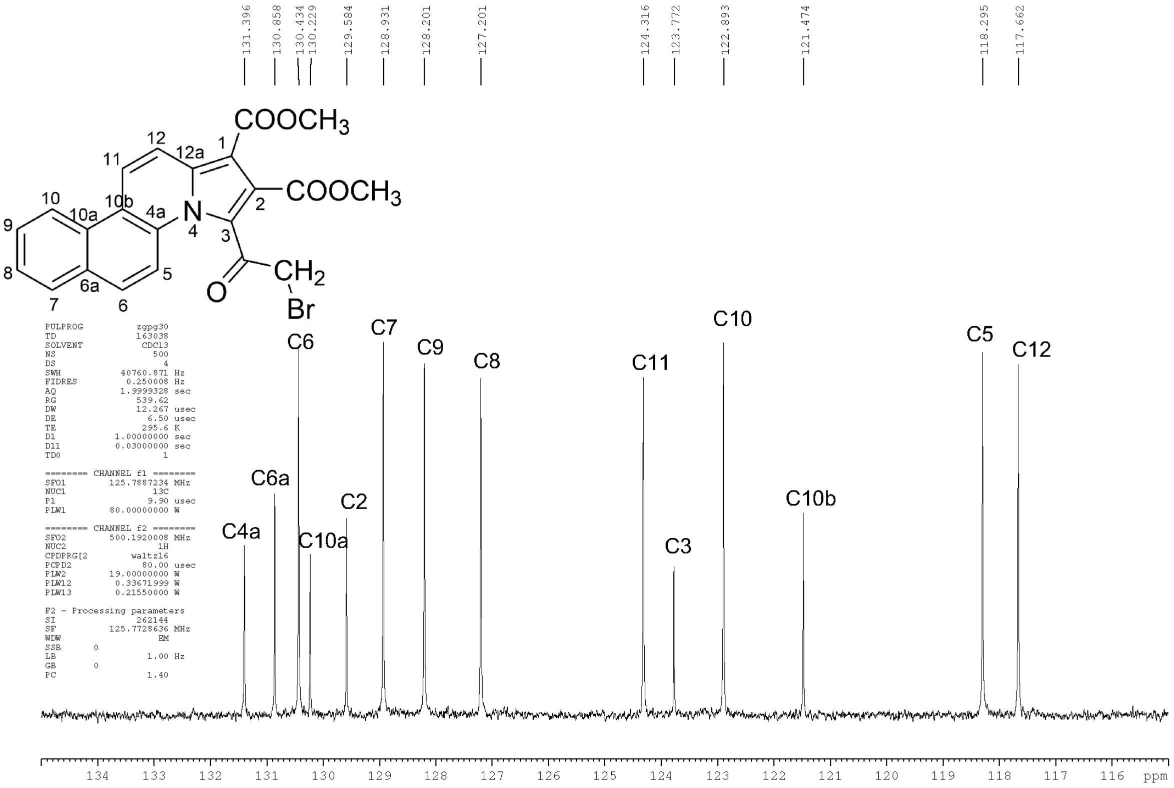

Figure.S2c Detail in the aromatic area of the ^{13}C NMR spectrum of the compound **2b.**

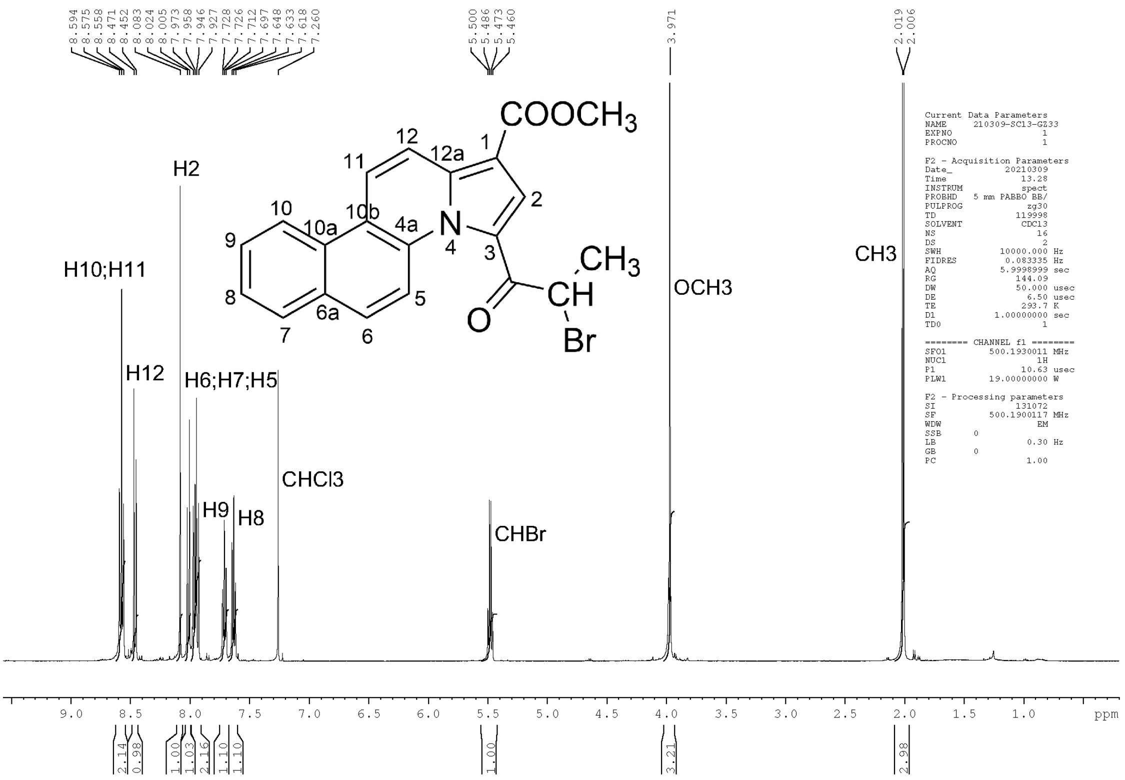

Figure S3a. ^{1}H NMR spectrum of the compound **2c.**

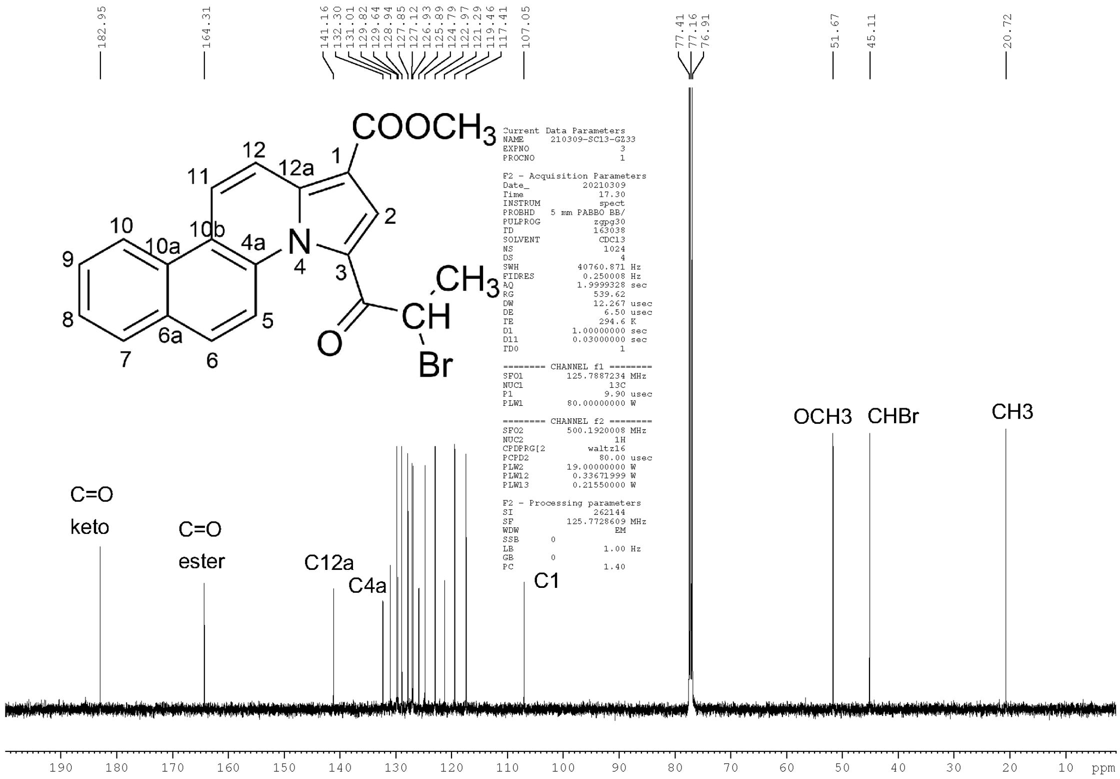

Figure S3b. ^{13}C NMR spectrum of the compound **2c.**

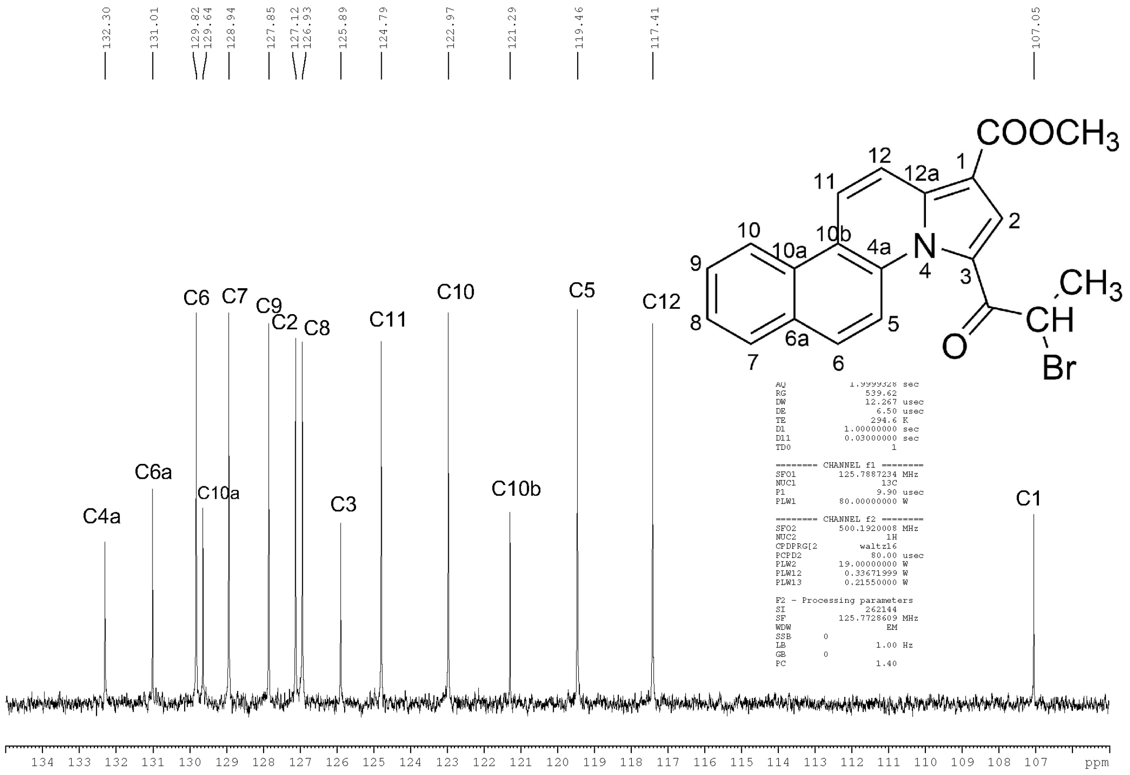

Figure S3c. Detail in the aromatic area of the ^{13}C NMR spectrum of the compound **2c.**

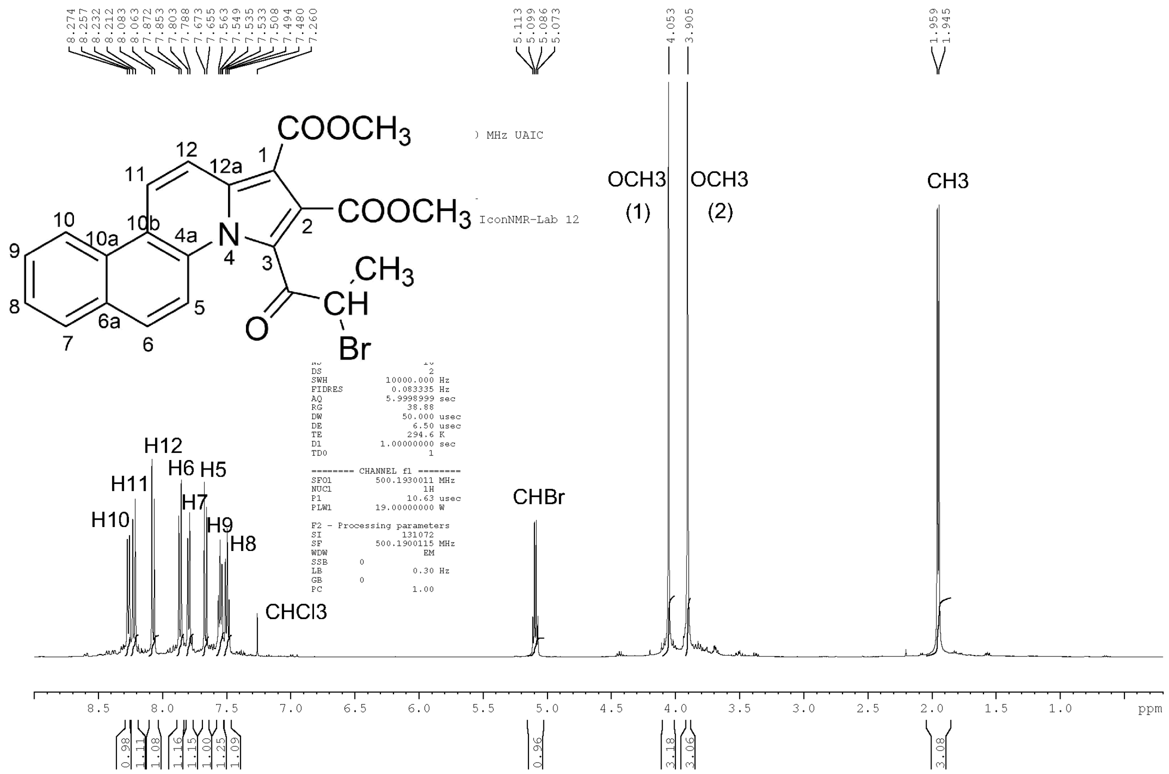

Figure S4a. ^{1}H NMR spectrum of the compound **2d.**

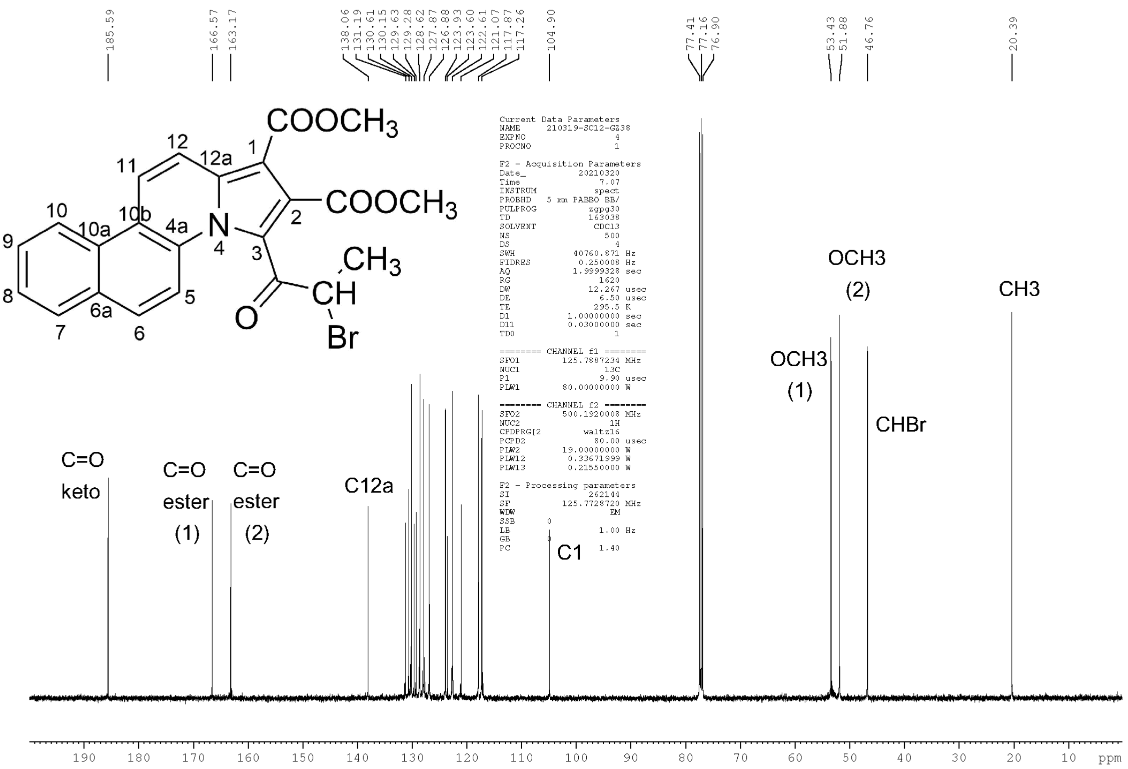

Figure S4b. ^{13}C NMR spectrum of the compound **2d.**

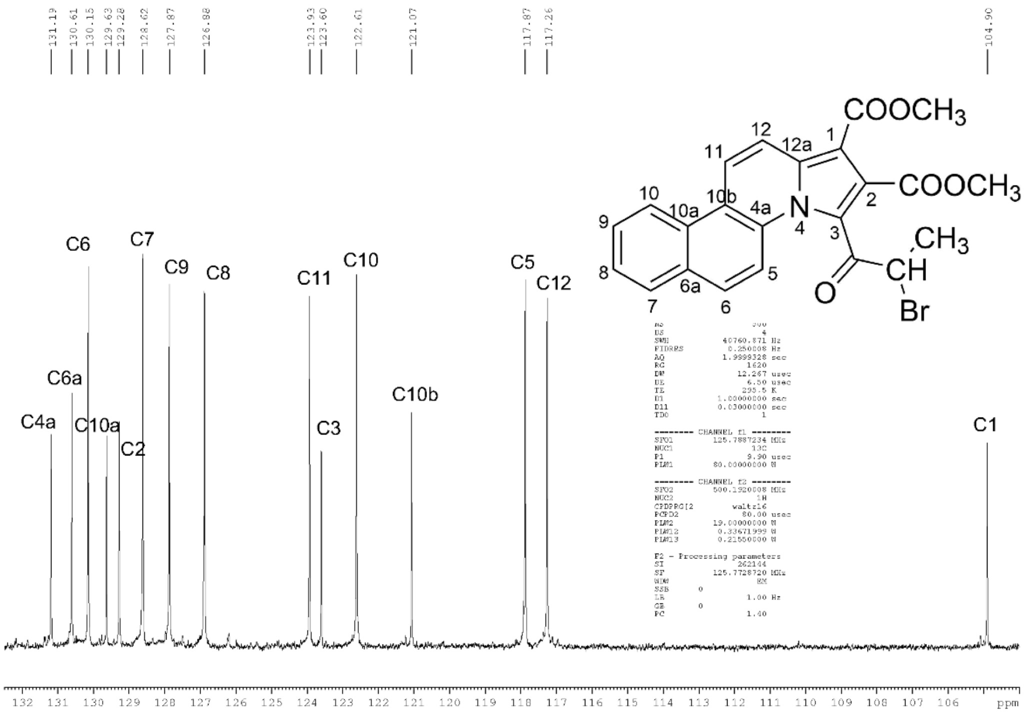

Figure S4c. Detail in the aromatic area of the ^{13}C NMR spectrum of the compound **2d.**

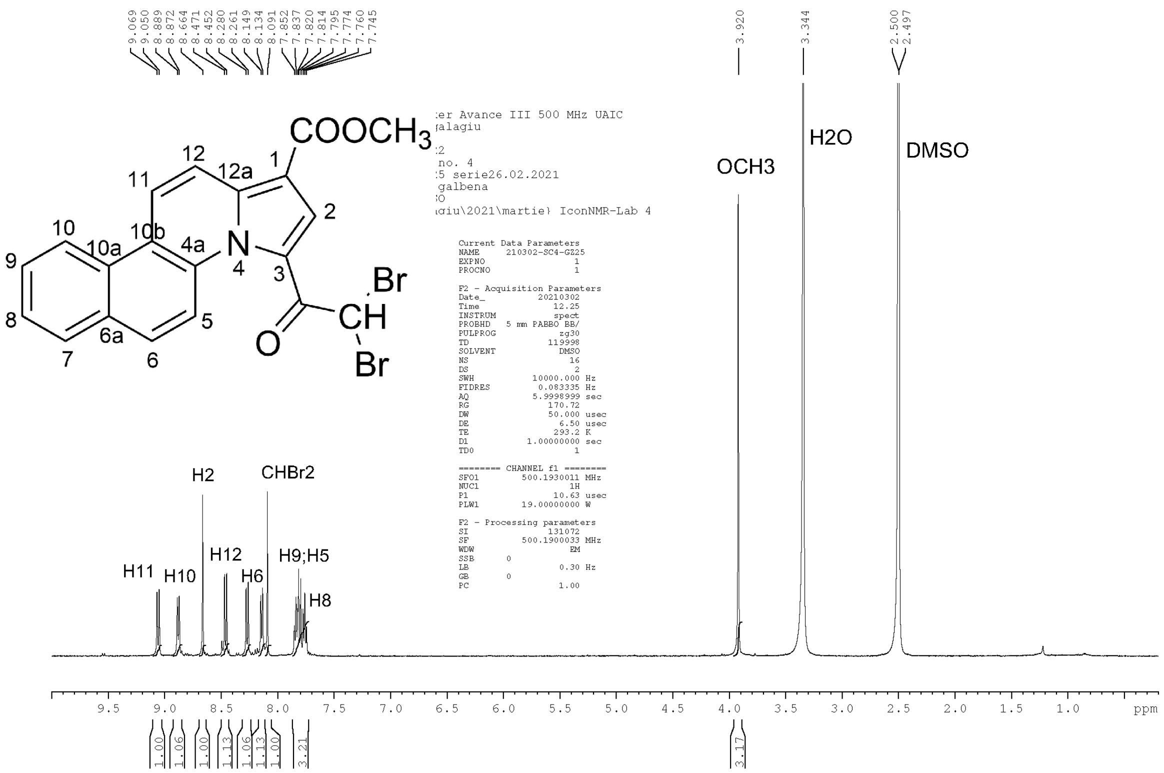

Figure S5a. ^{1}H NMR spectrum of the compound **3a.**

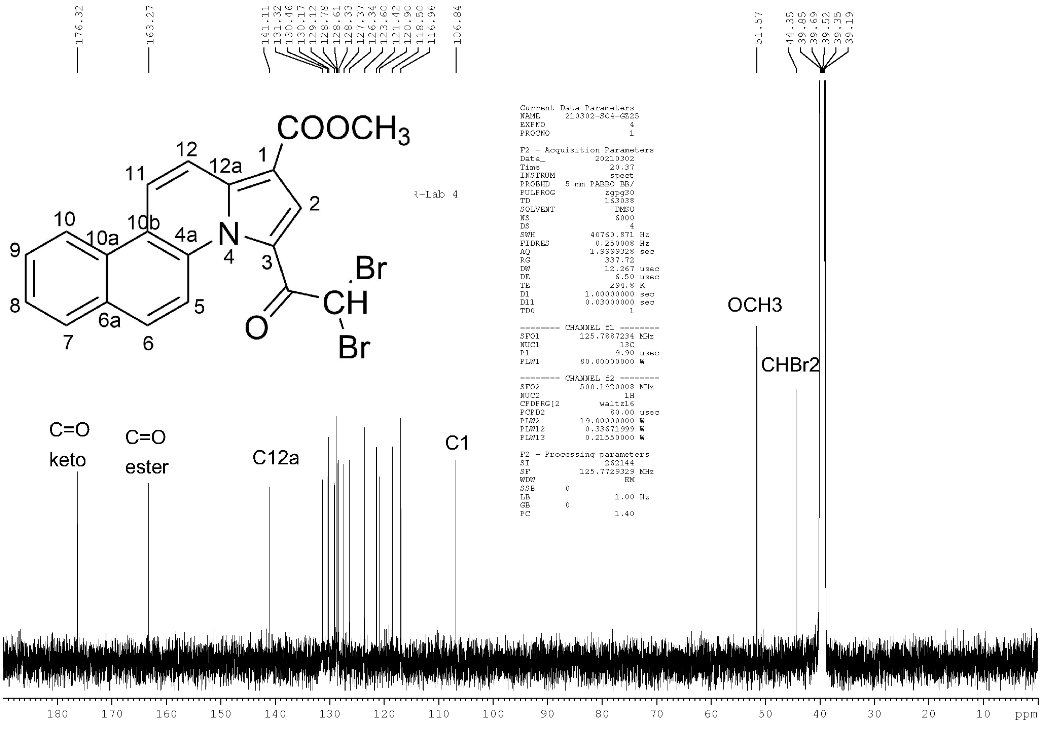

Figure S5b. ^{13}C NMR spectrum of the compound **3a.**

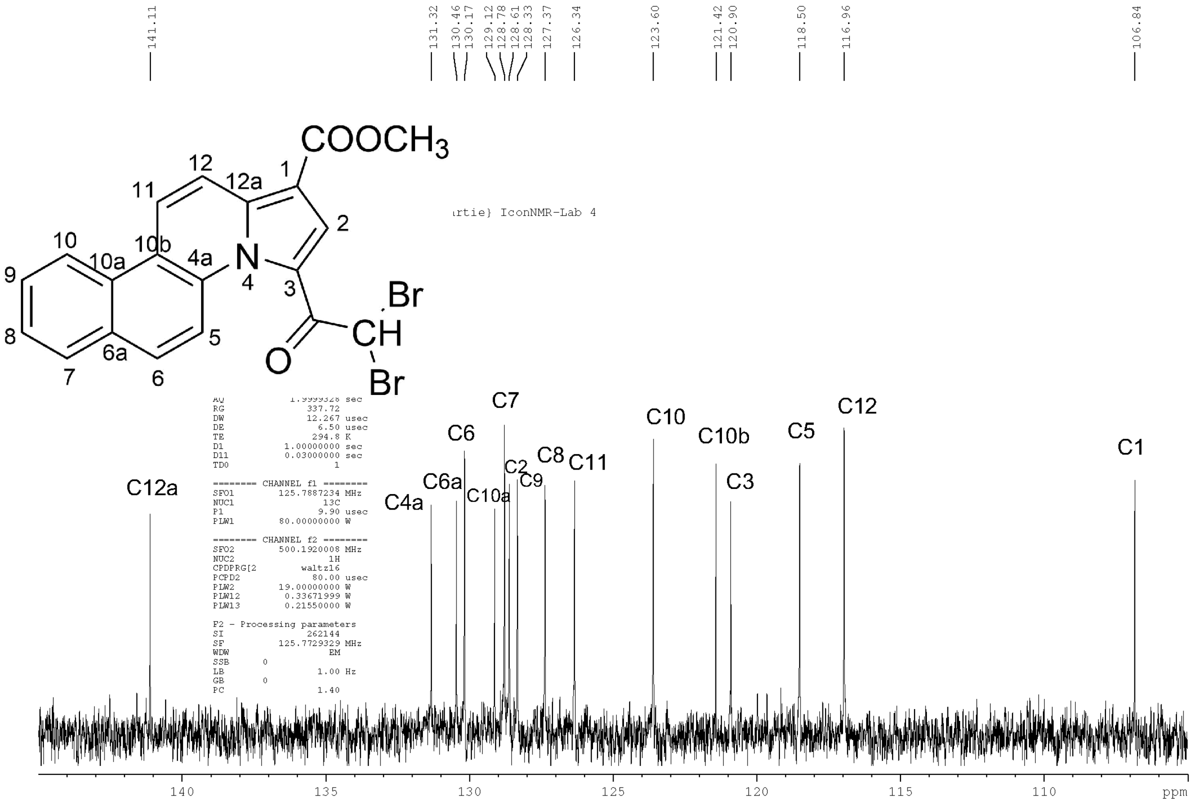

Figure S5c. Detail in the aromatic area of the ^{13}C NMR spectrum of the compound **3a.**

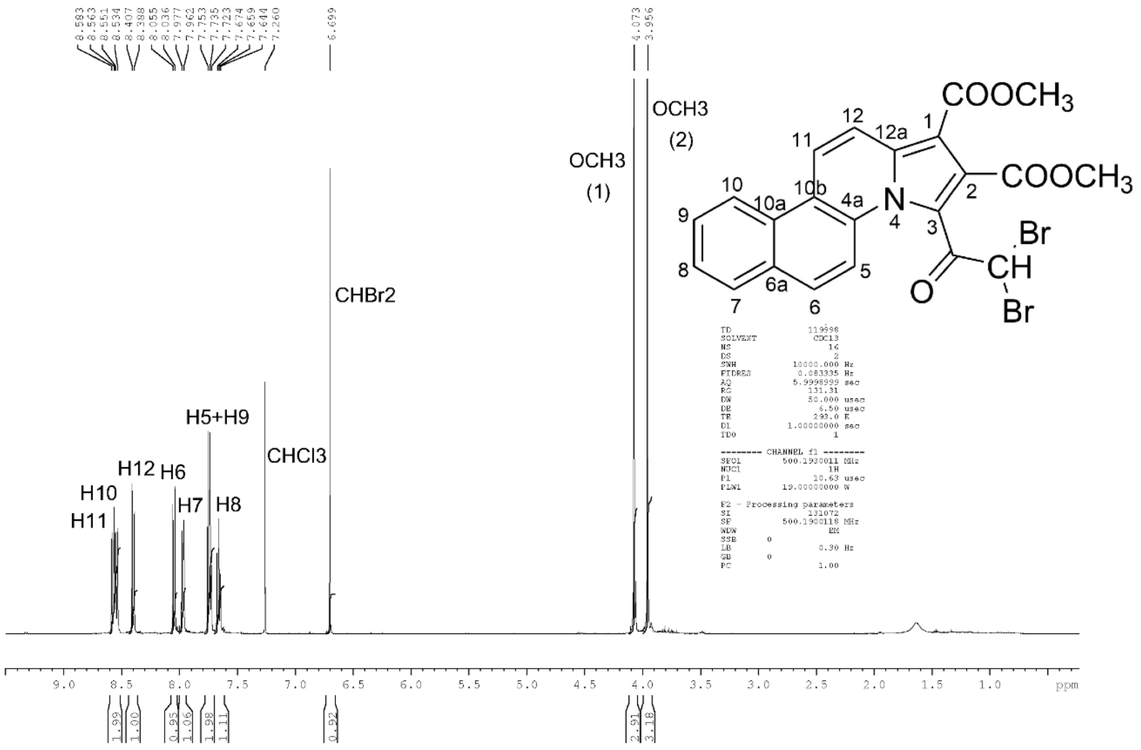

Figure S6a. ^{1}H NMR spectrum of the compound **3b.**

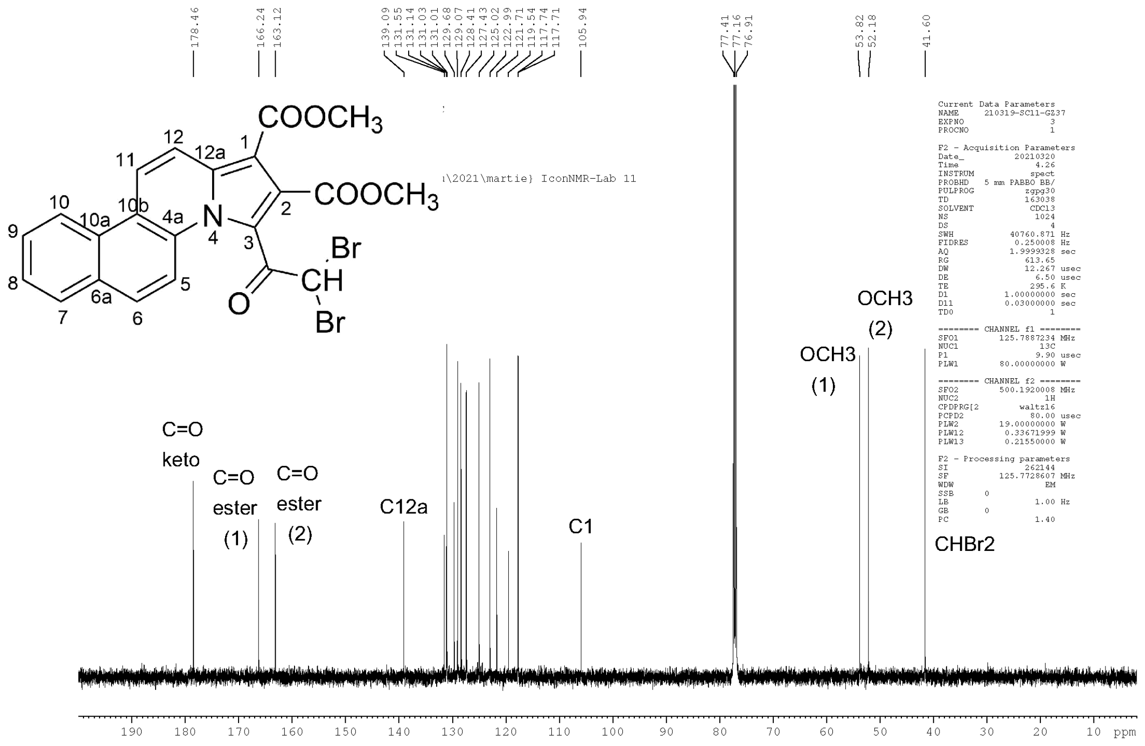

Figure S6b. ^{13}C NMR spectrum of the compound **3b.**

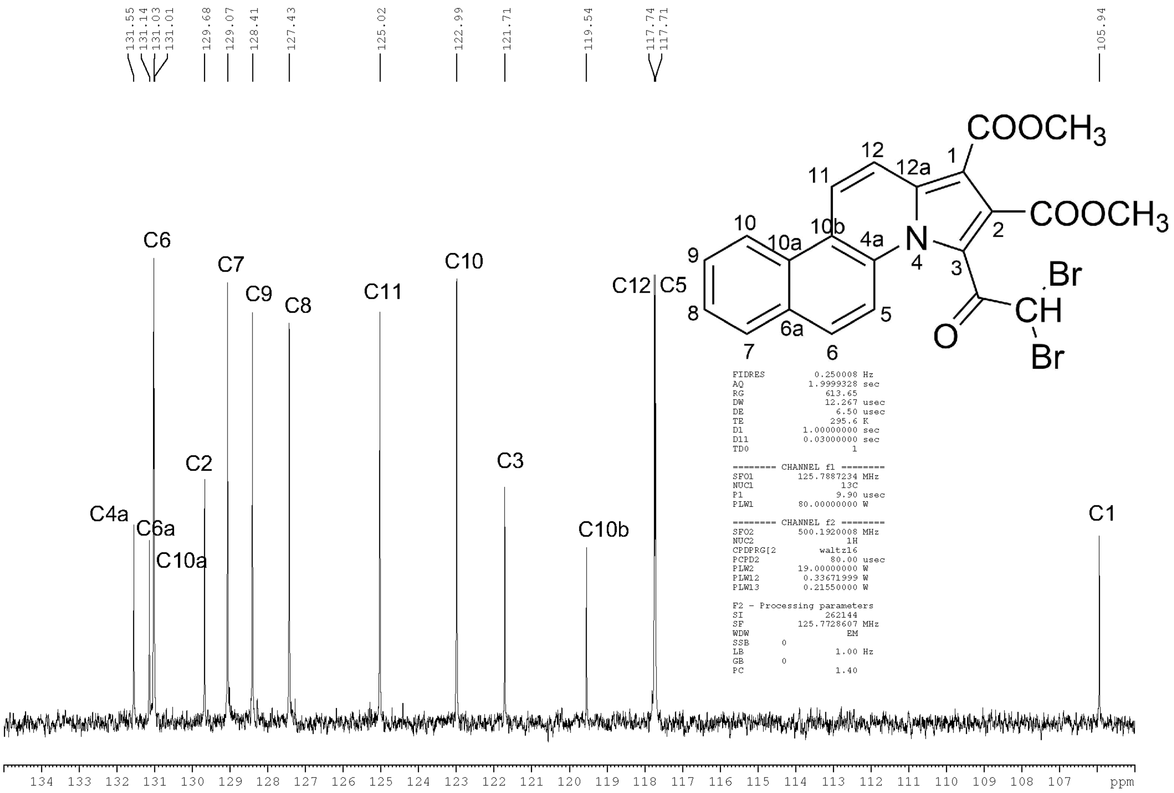

Figure S6c. Detail in the aromatic area of the ^{13}C NMR spectrum of the compound **3b.**

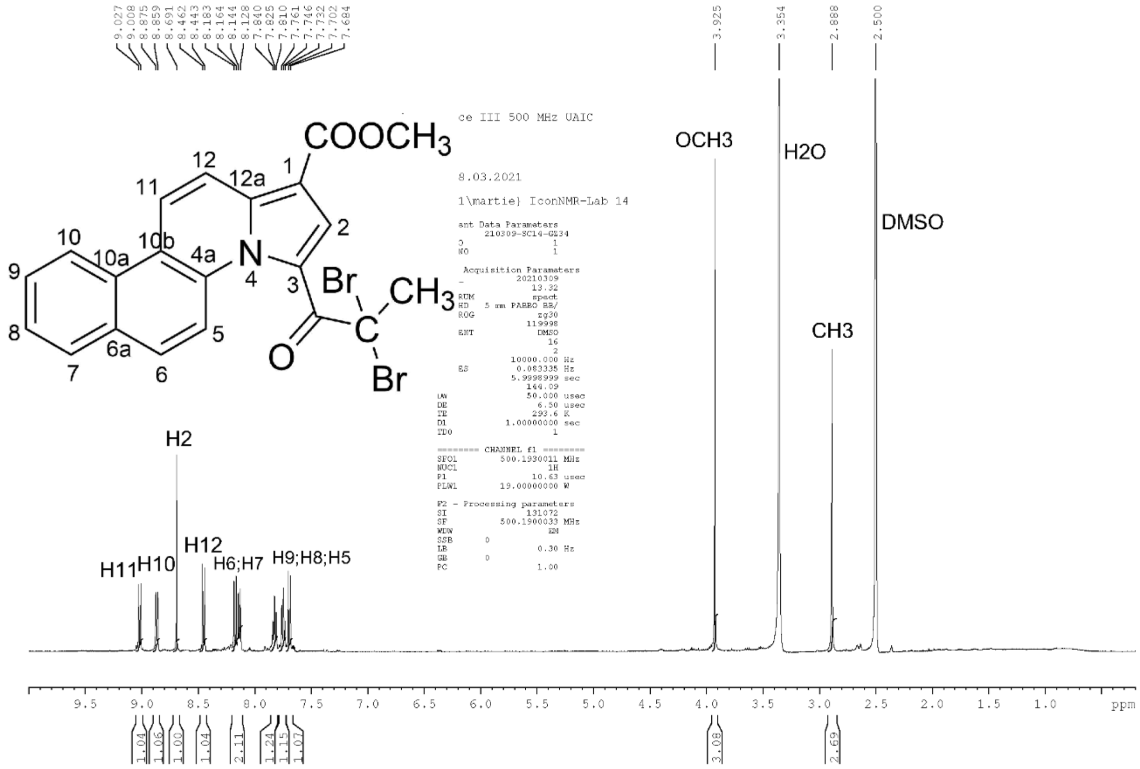

Figure S7a. ^{1}H NMR spectrum of the compound **3c.**

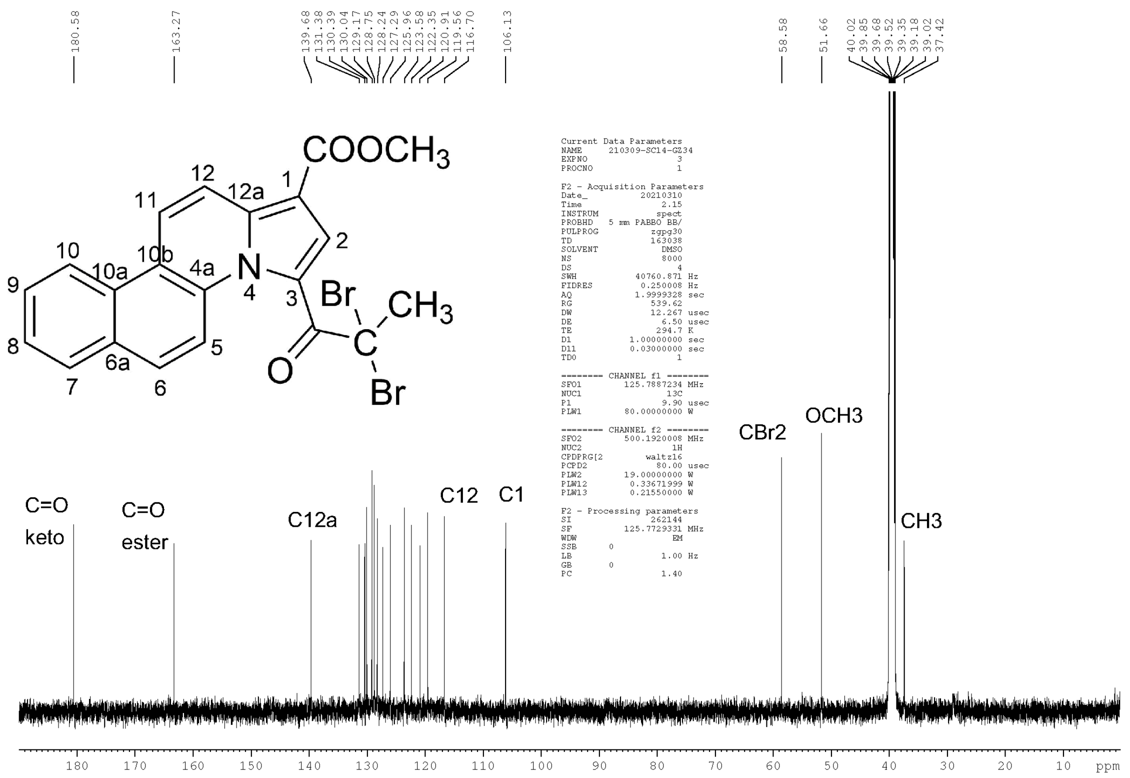

Figure S7b. ^{13}C NMR spectrum of the compound **3c.**

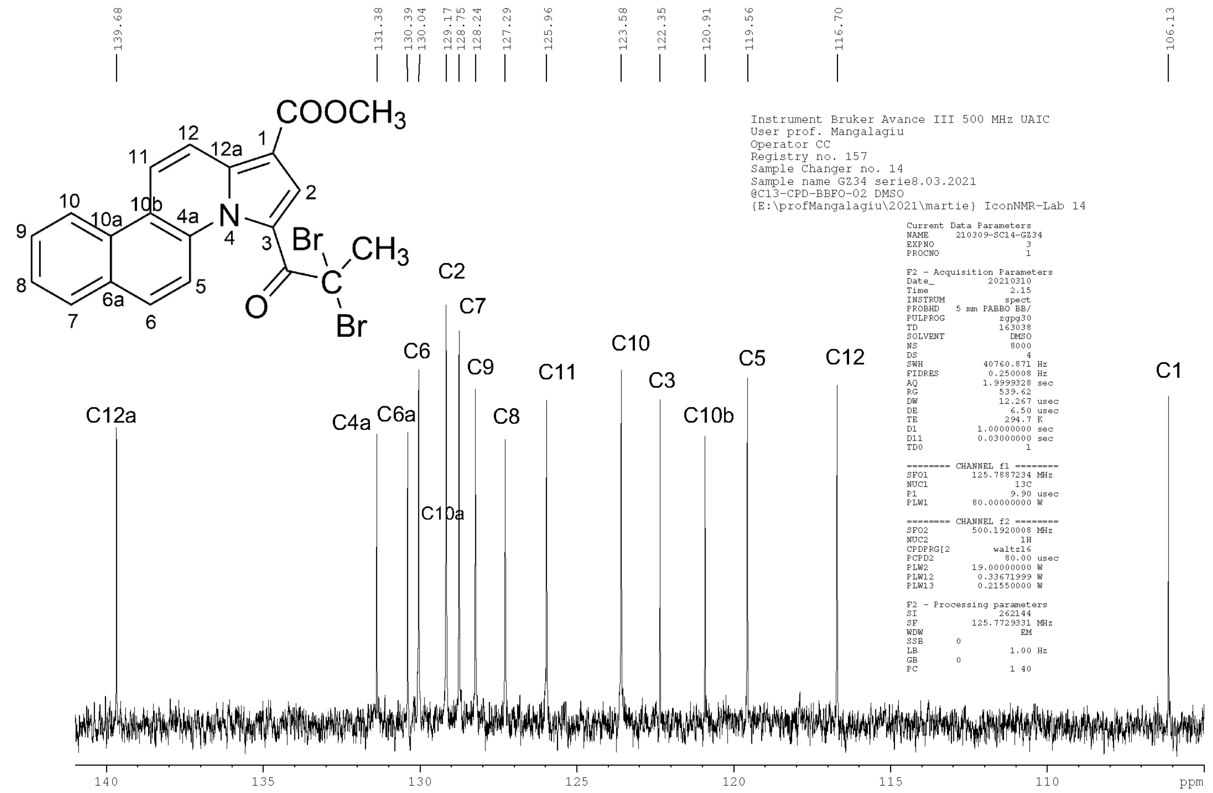

Figure S7c. Detail in the aromatic area of the ^{13}C NMR spectrum of the compound **3c.**

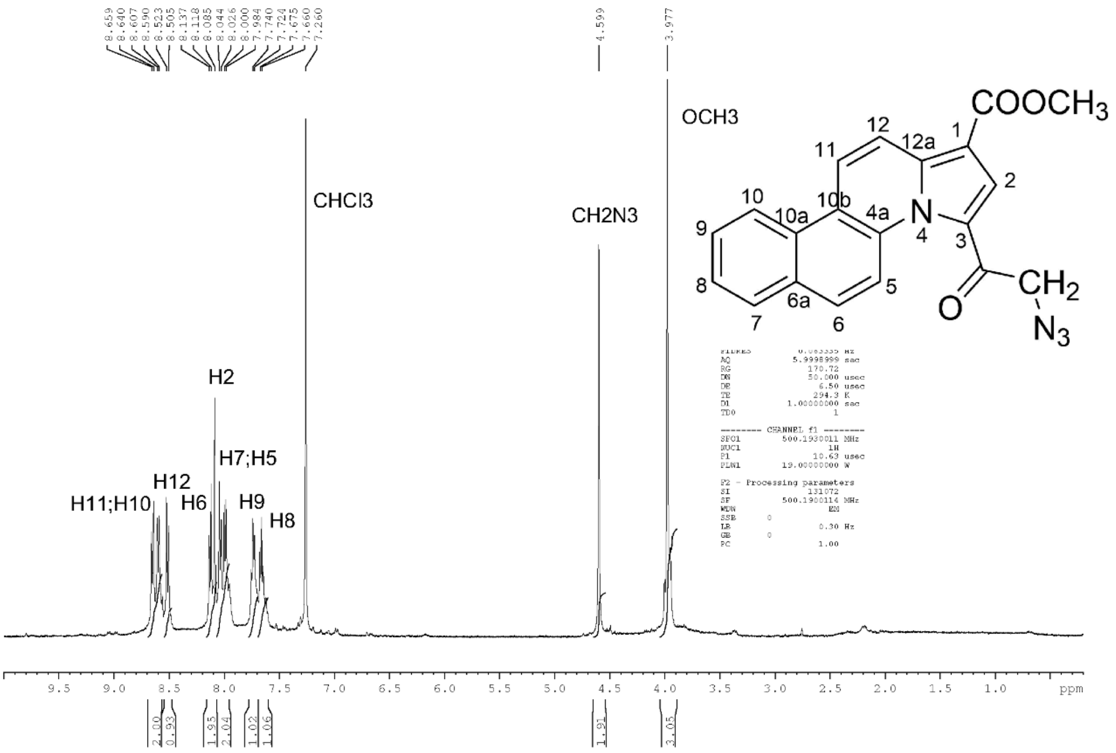

Figure S8a. ^{1}H NMR spectrum of the compound **4a.**

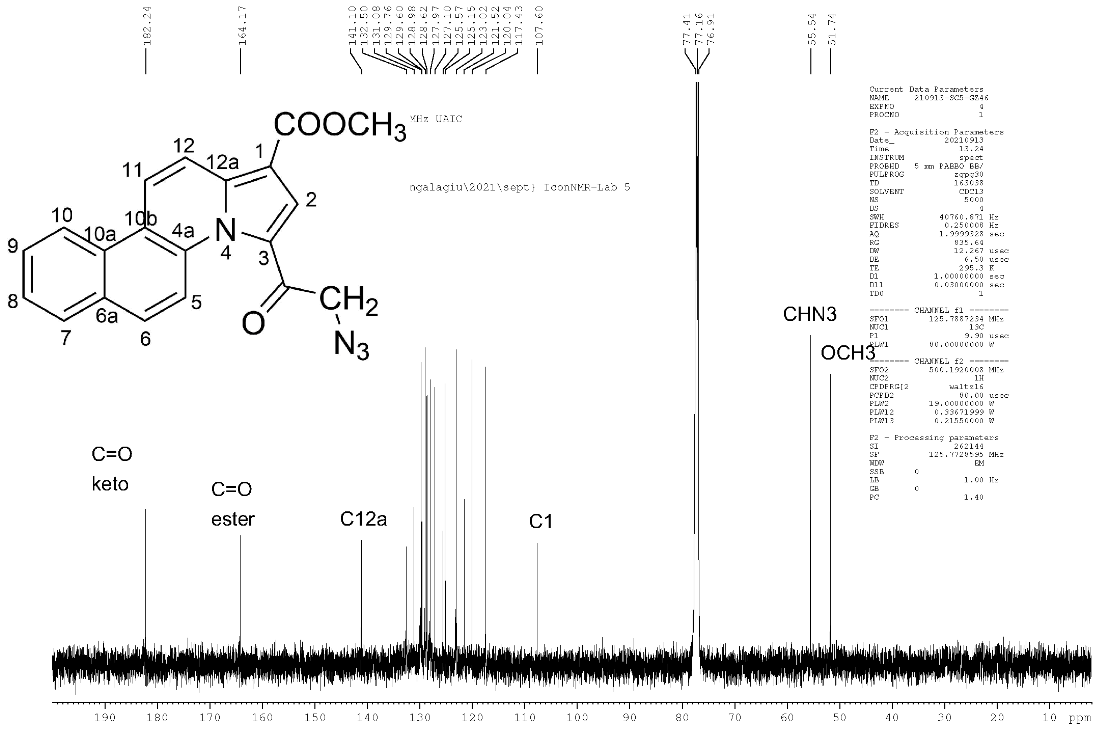

Figure S8b. ^{13}C NMR spectrum of the compound **4a.**

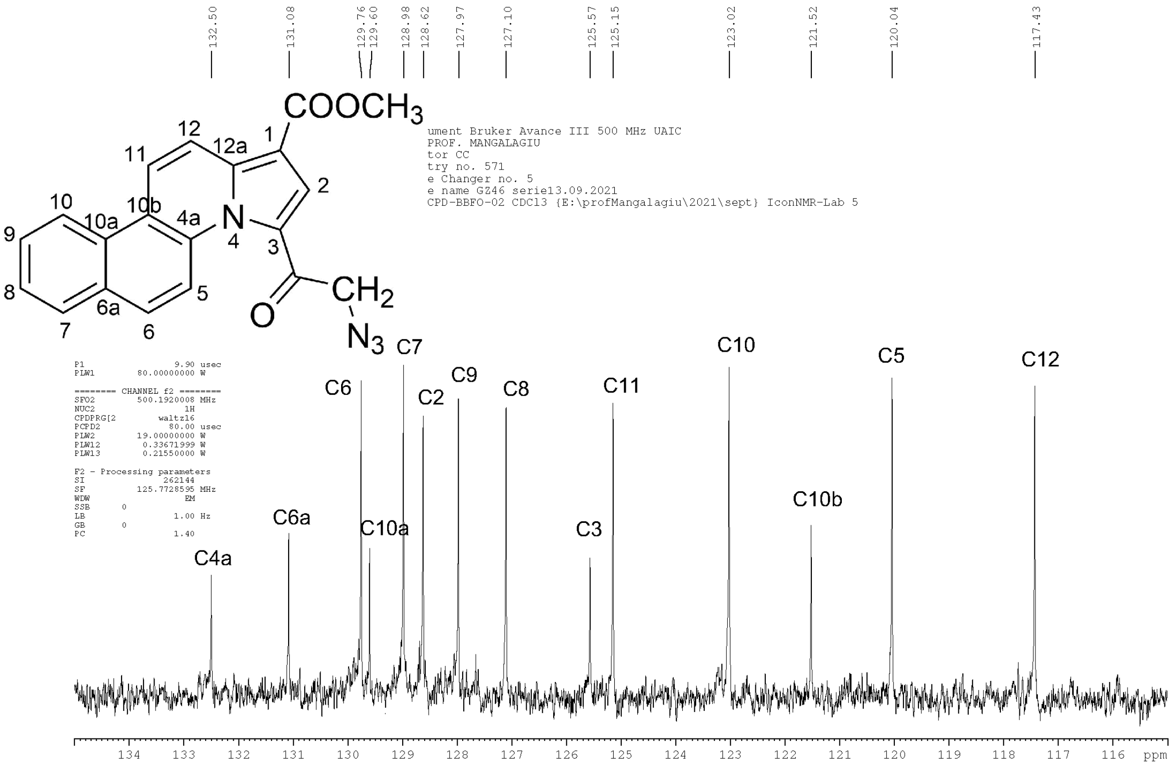

Figure S8c. Detail in the aromatic area of the ^{13}C NMR spectrum of the compound **4a.**

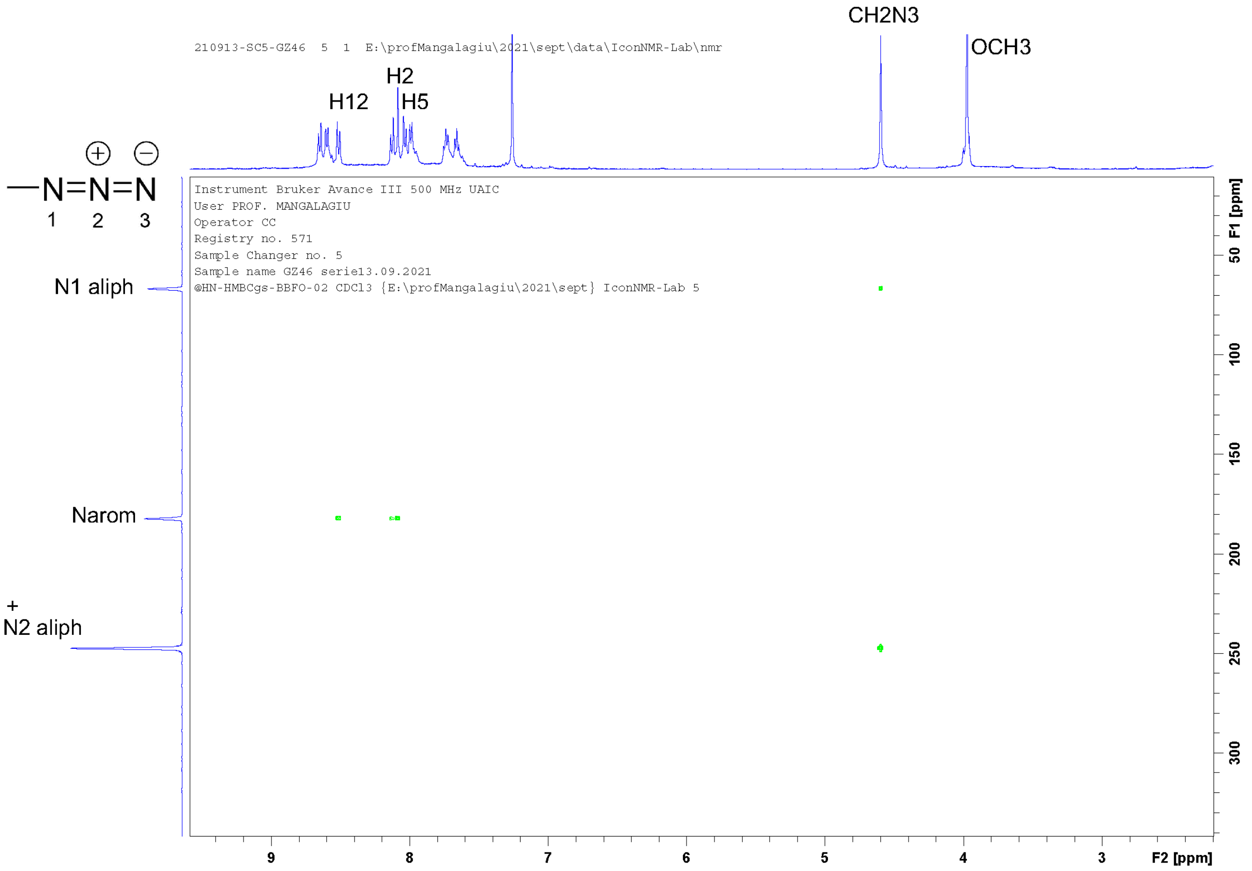

Figure S8d. Long range ^{1}H-^{15}N correlation HMBC spectrum of the compound **4a.**

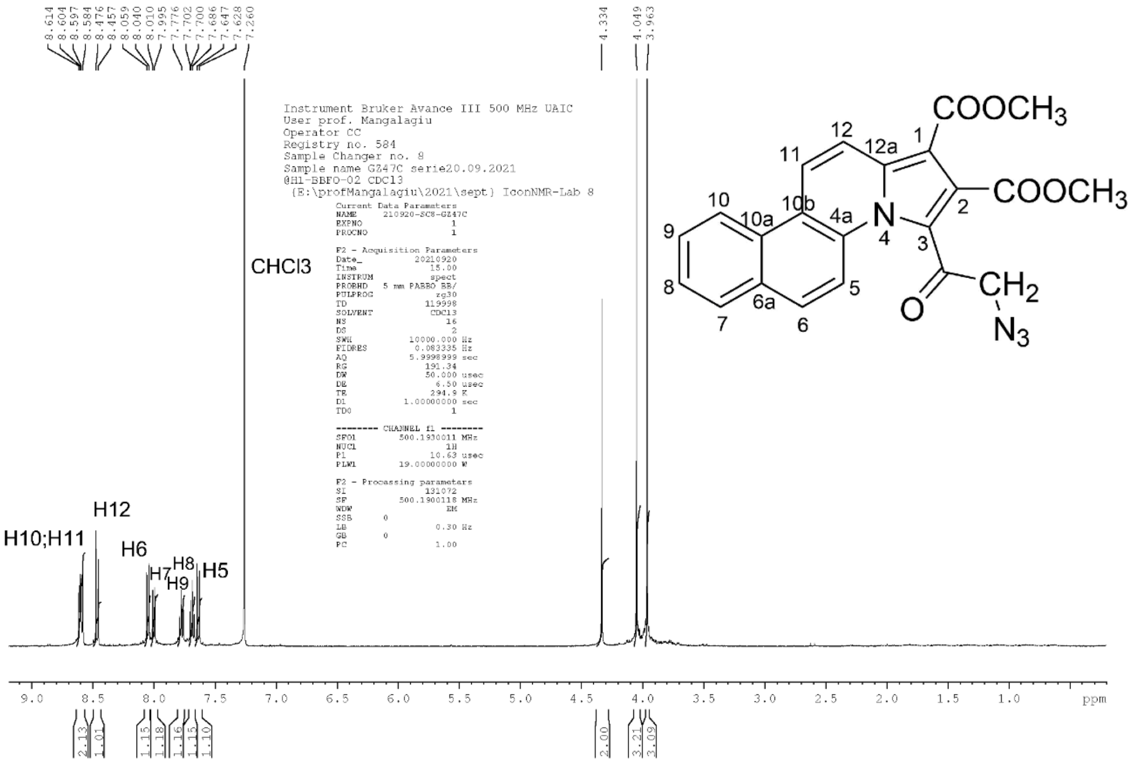

Figure S9a. ^{1}H NMR spectrum of the compound **4b**.

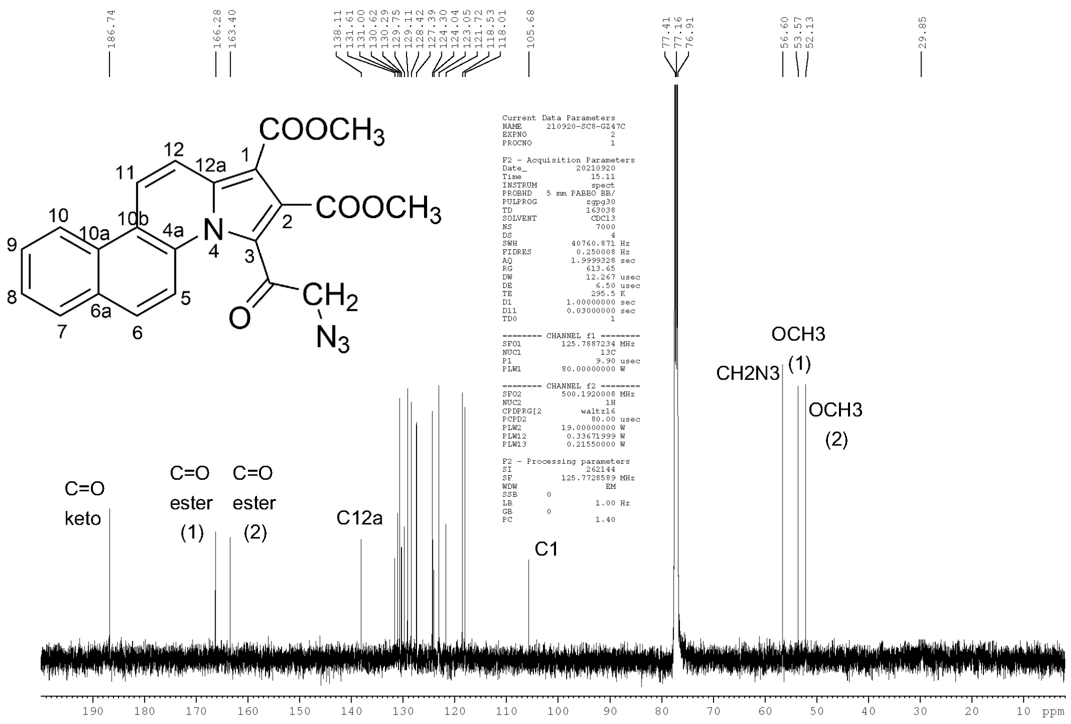

Figure S9b. ^{13}C NMR spectrum of the compound **4b**.

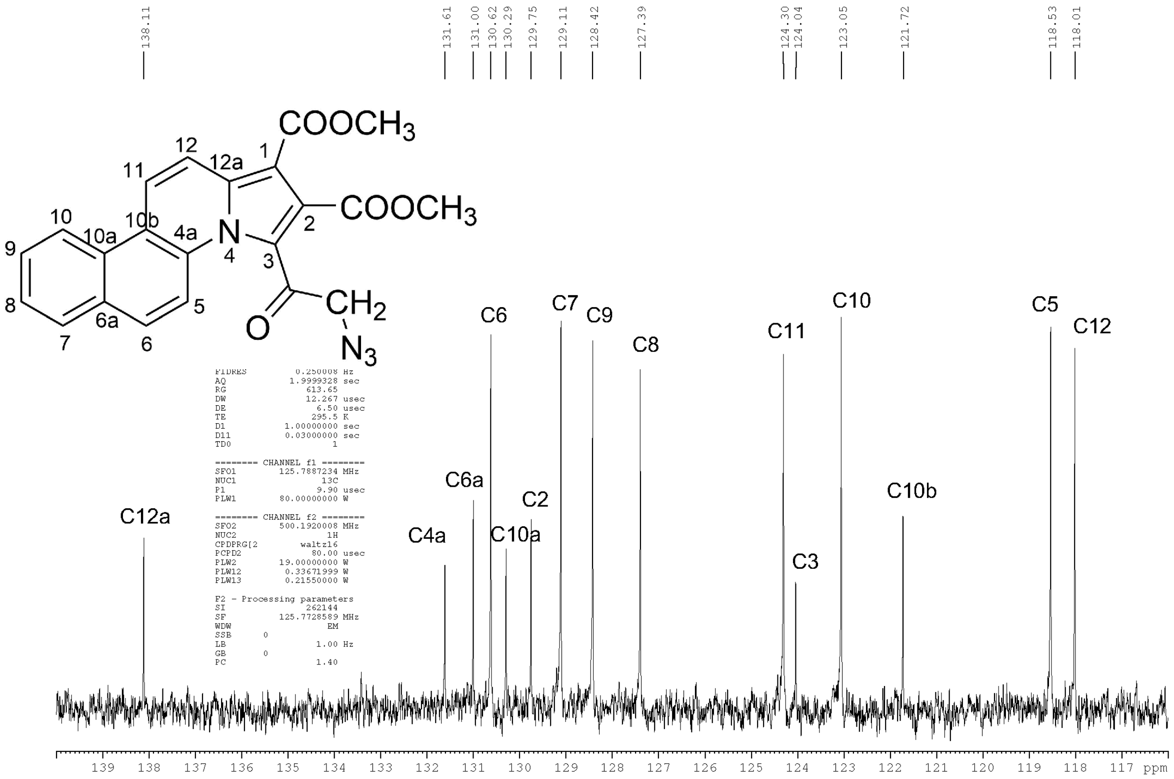

Figure S9c . Detail in the aromatic area of the ^{13}C NMR spectrum of the compound **4b.**

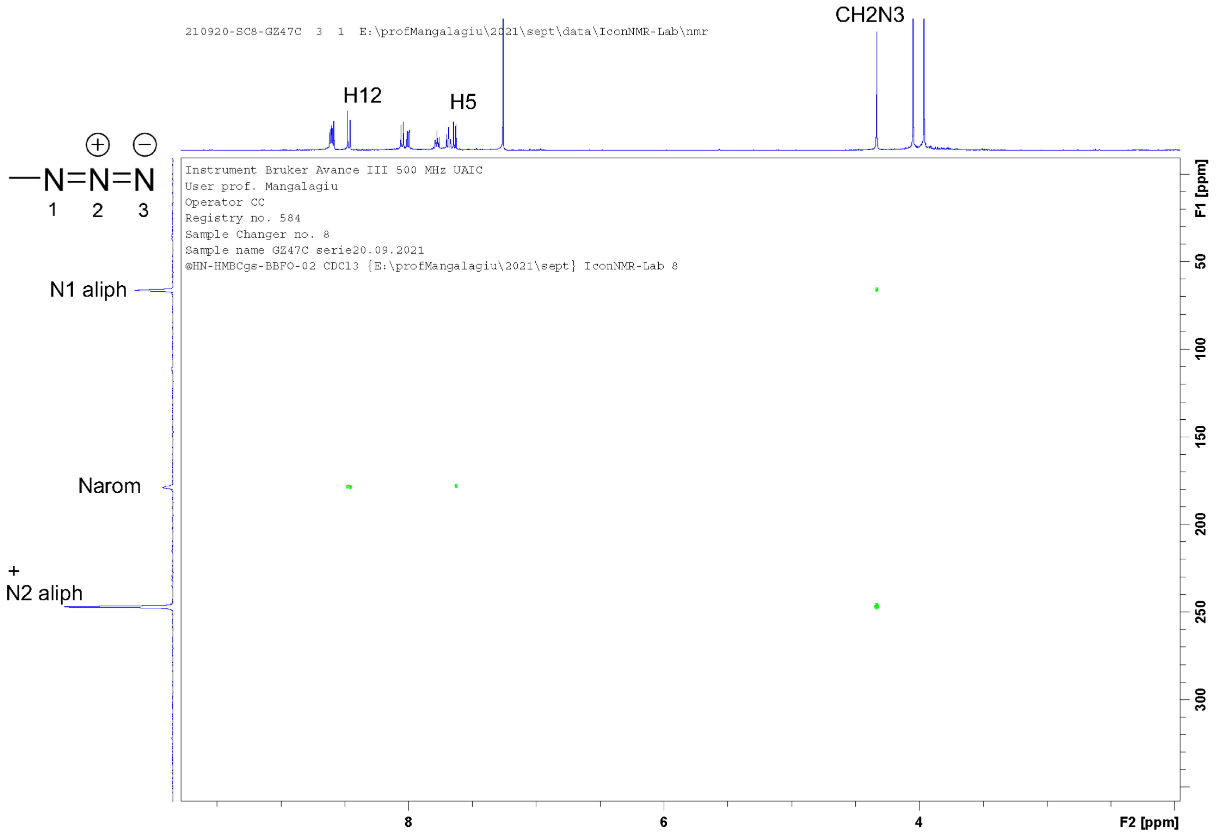

Figure S9d. Long range ^{1}H-^{15}N correlation HMBC spectrum of the compound **4b.**

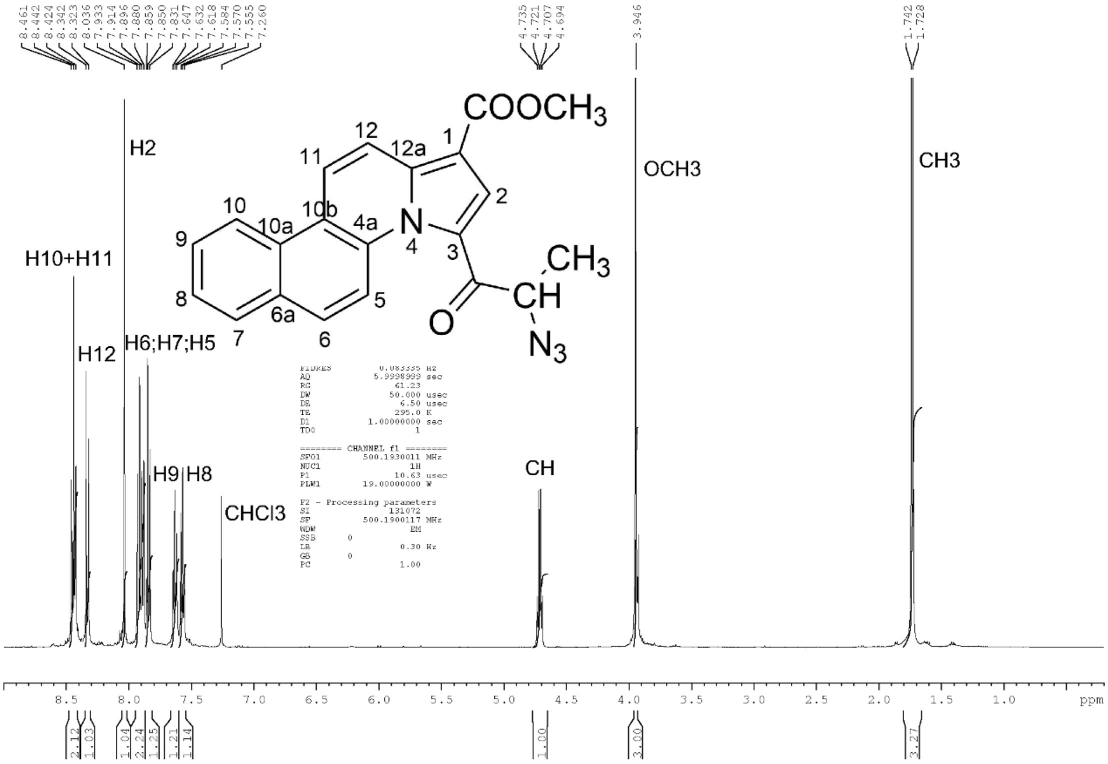

Figure S10a. ^{1}H NMR spectrum of the compound **4c.**

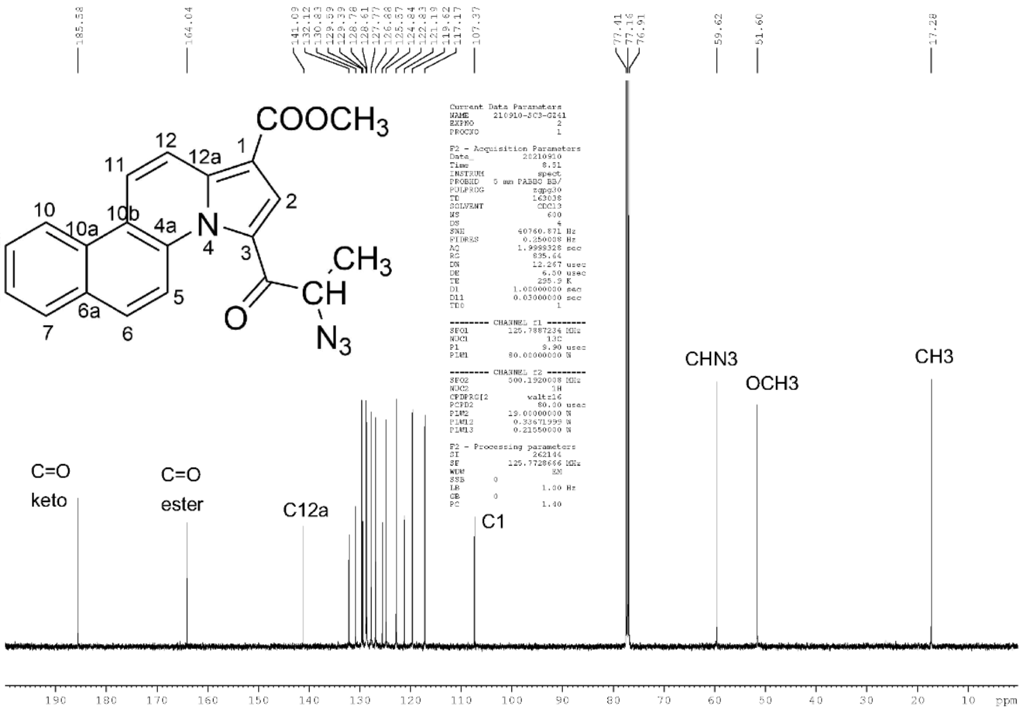

Figure S10b. ^{13}C NMR spectrum of the compound **4c.**

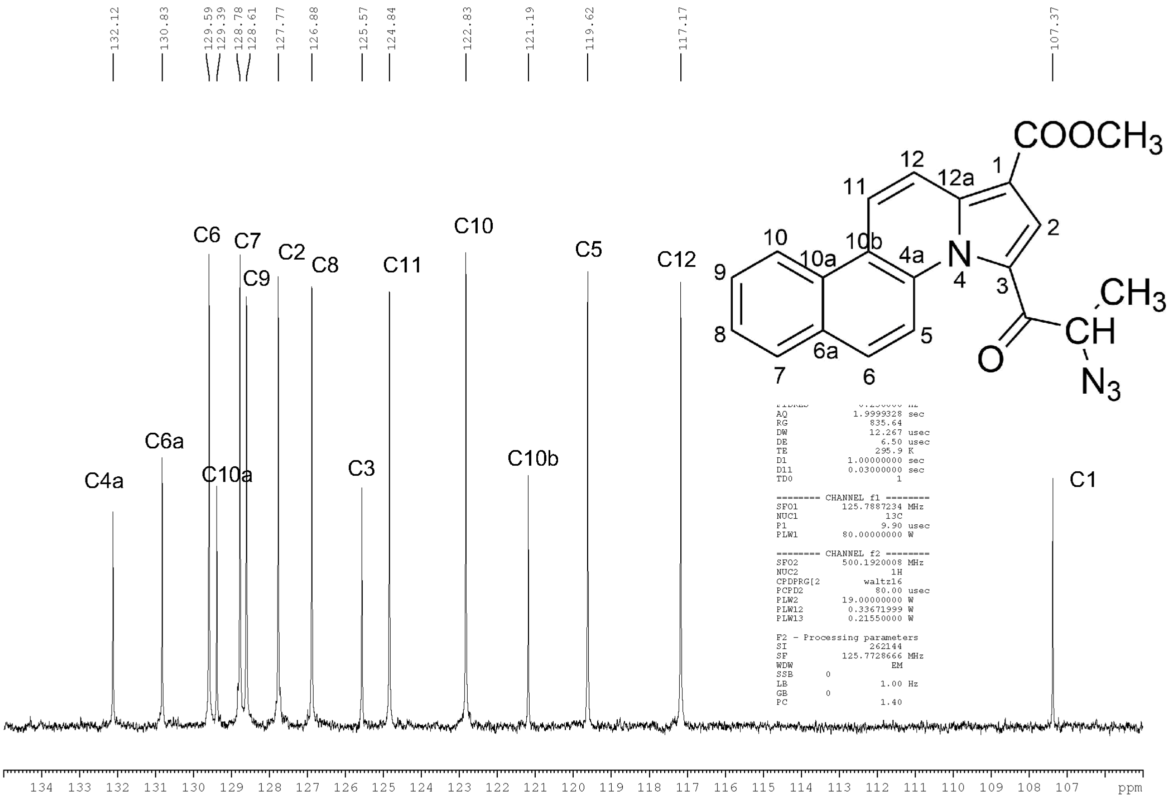

Figure S10c. Detail in the aromatic area of the ^{13}C NMR spectrum of the compound **4c.**

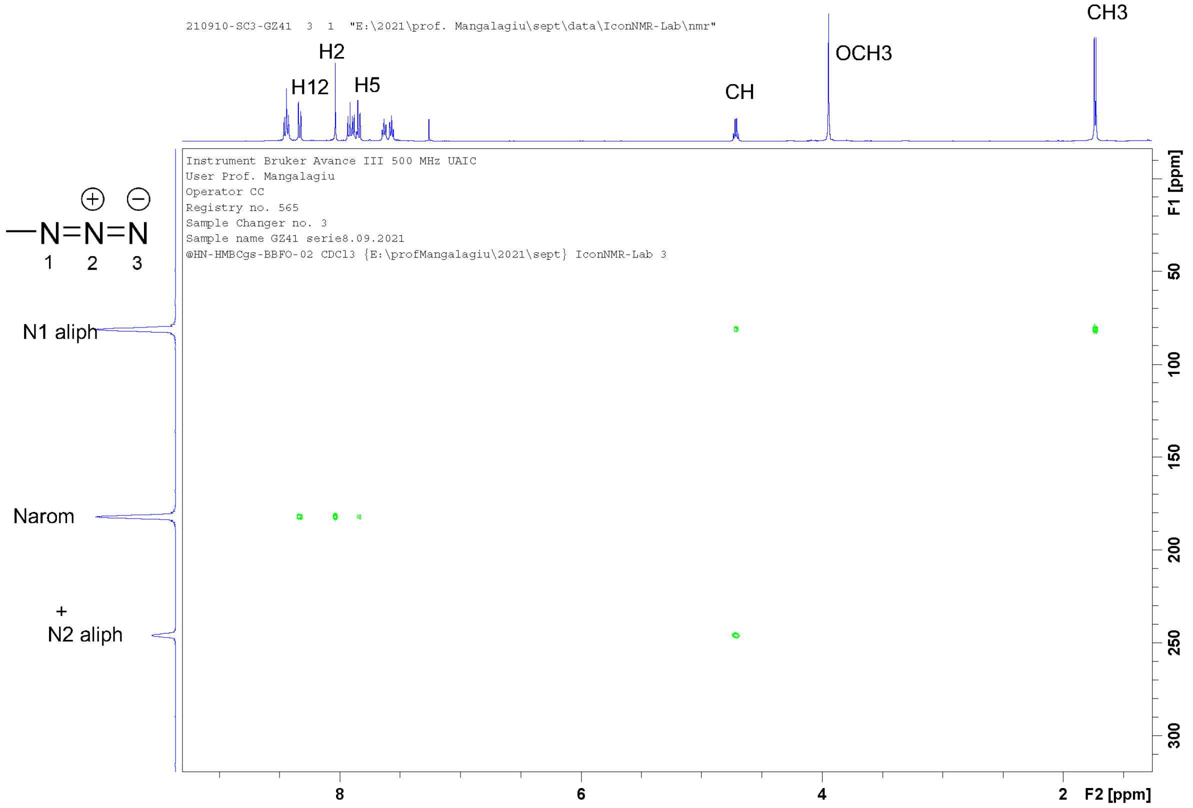

Figure S10d . Long range ^{1}H-^{15}N correlation HMBC spectrum of the compound **4c.**

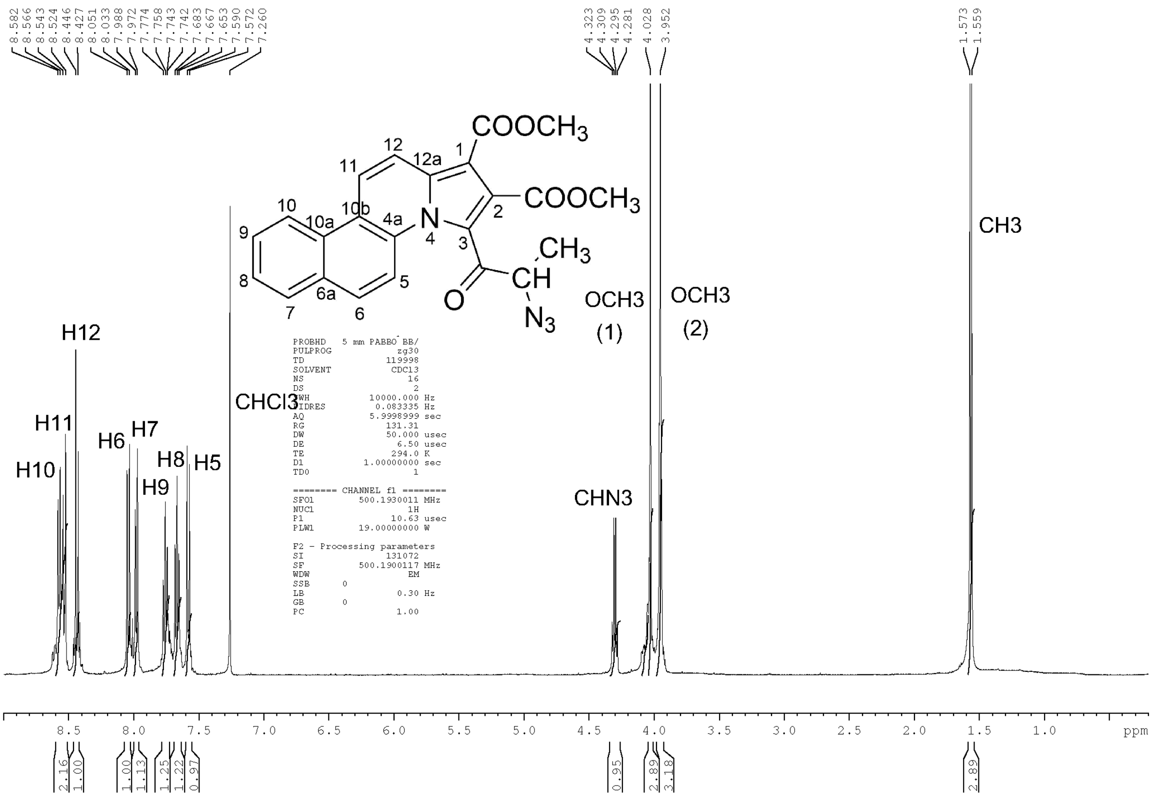

Figure S11a. ^{1}H NMR spectrum of the compound **4d.**

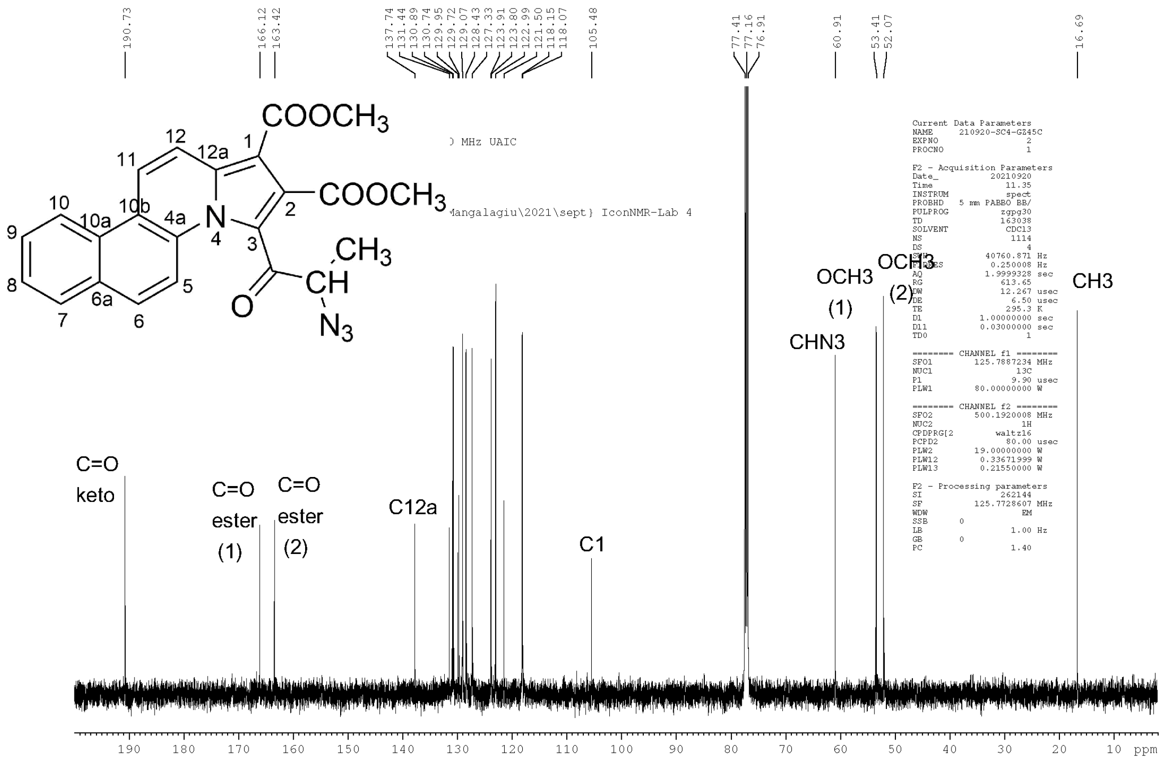

Figure S11b. ^{13}C NMR spectrum of the compound **4d.**

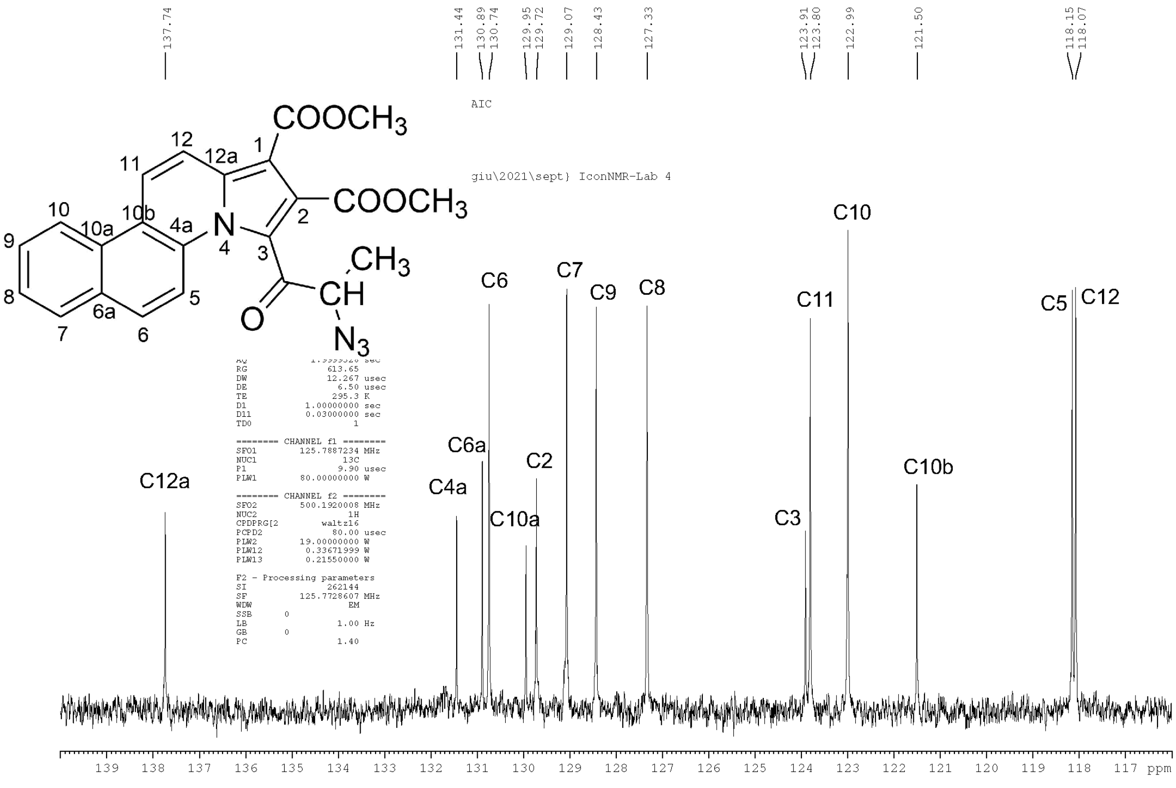

Figure S11c. Detail in the aromatic area of the ^{13}C NMR spectrum of the compound **4d.**

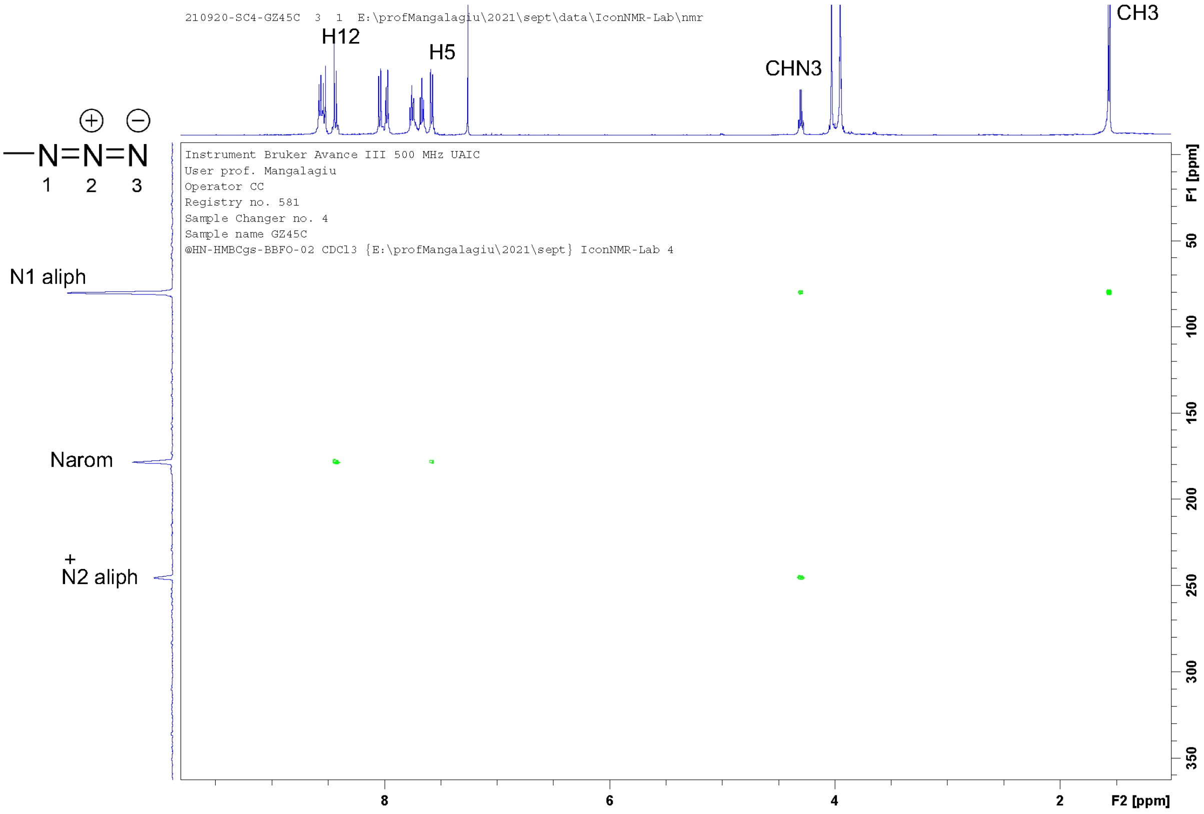

Figure S11d. Long range ^{1}H-^{15}N correlation HMBC spectrum of the compound **4d.**

3. IR spectra of the obtained compounds

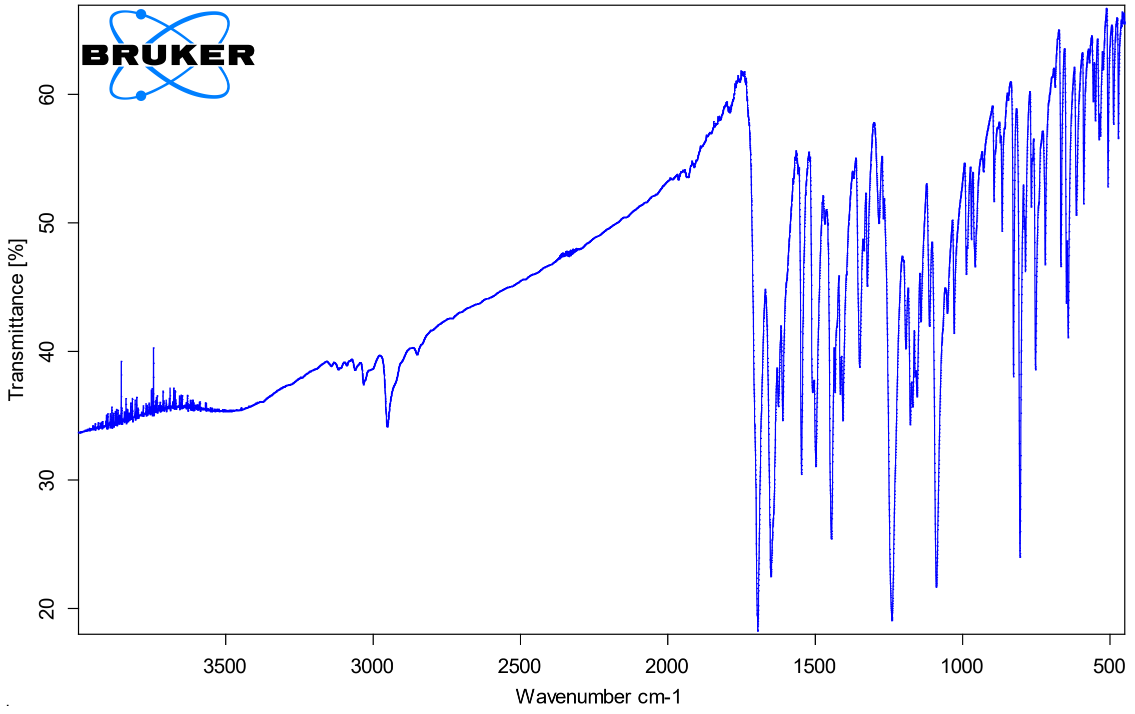

C:\OPUS_7.0.129\WORK\2021-09-28\2021-09-28-2A.0 TEST Instrument type and / or accessory	28/09/2021

Page 1/1

Figure S12. IR spectrum of the compound **2a.**

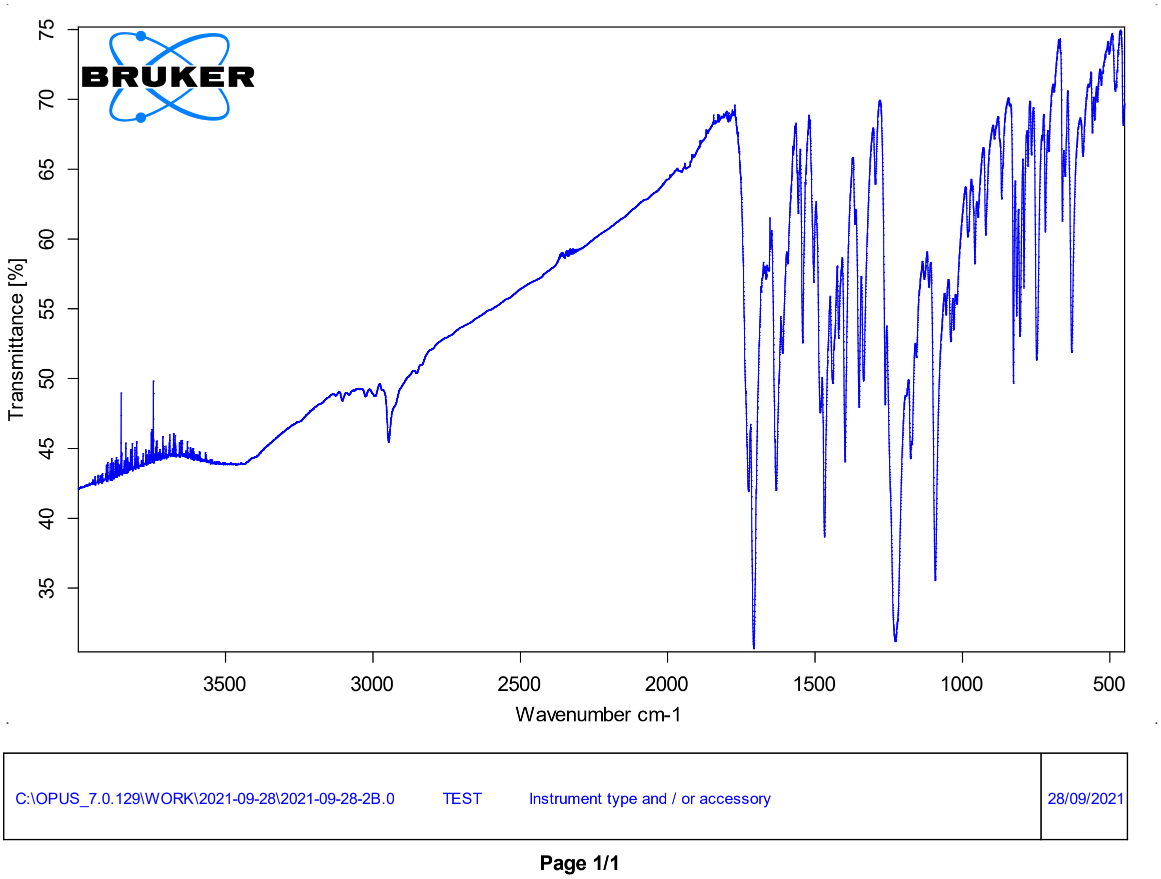

Figure S13. IR spectrum of the compound **2b.**

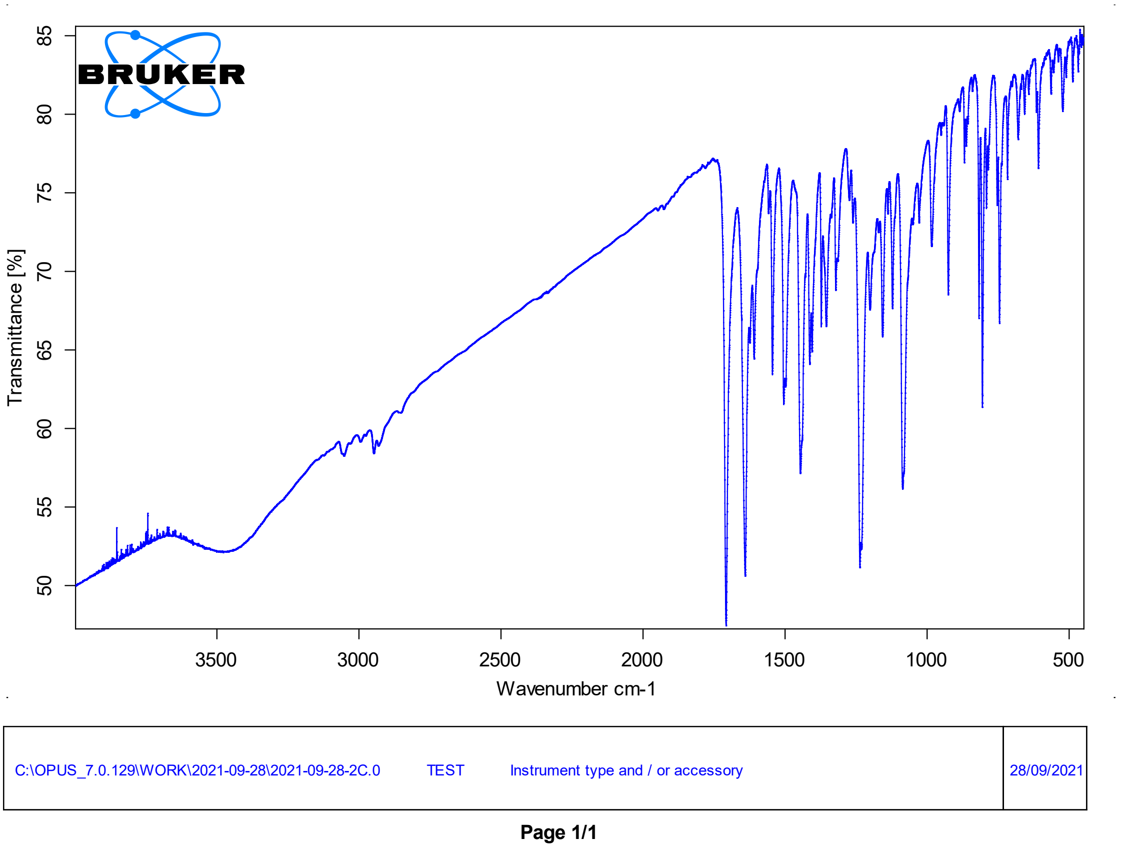

Figure S14. IR spectrum of the compound **2c.**

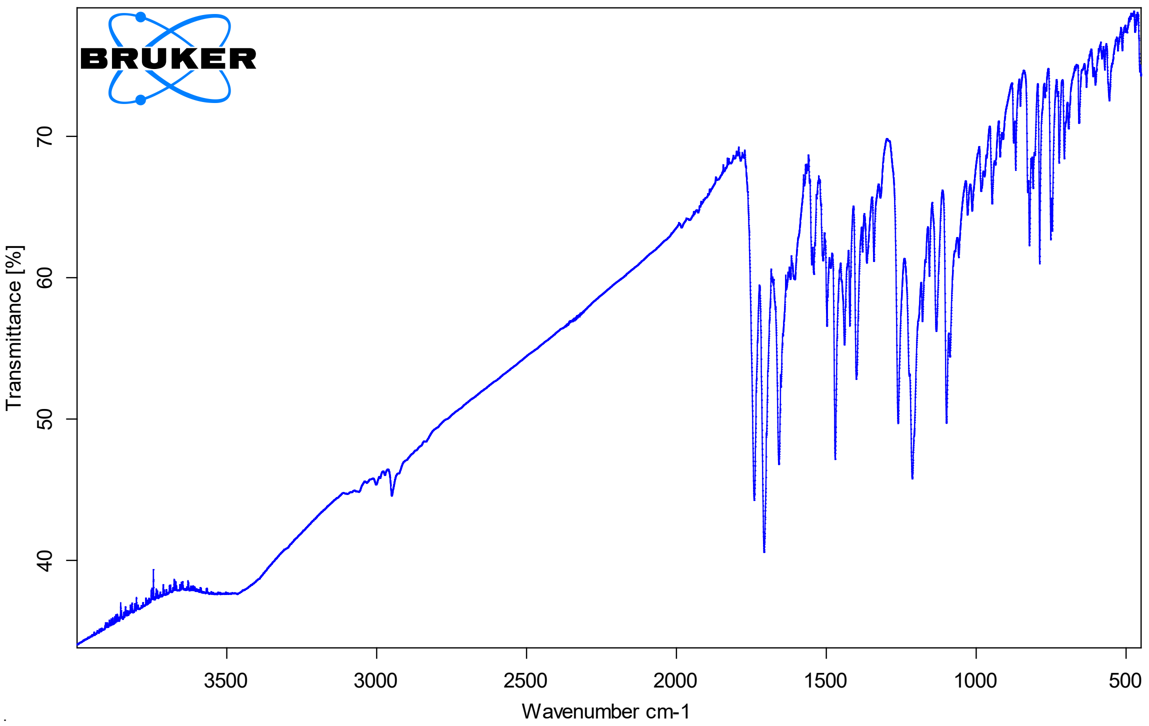

C:\OPUS_7.0.129\WORK\2021-09-28\2021-09-28-2D.0 TEST Instrument type and / or accessory	28/09/2021

Page 1/1

Figure S15. IR spectrum of the compound **2d.**

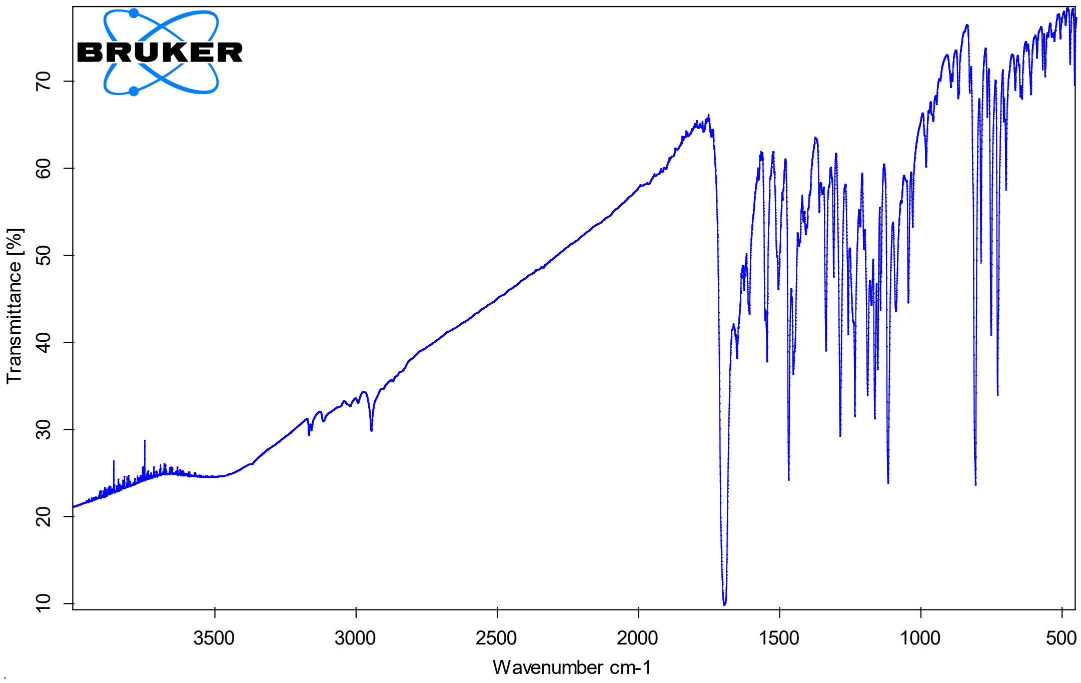

C:\OPUS_7.0.129\WORK\2021-09-28\2021-09-28-3A.0 TEST Instrument type and / or accessory	28/09/2021

Page 1/1

Figure S16. IR spectrum of the compound **3a.**

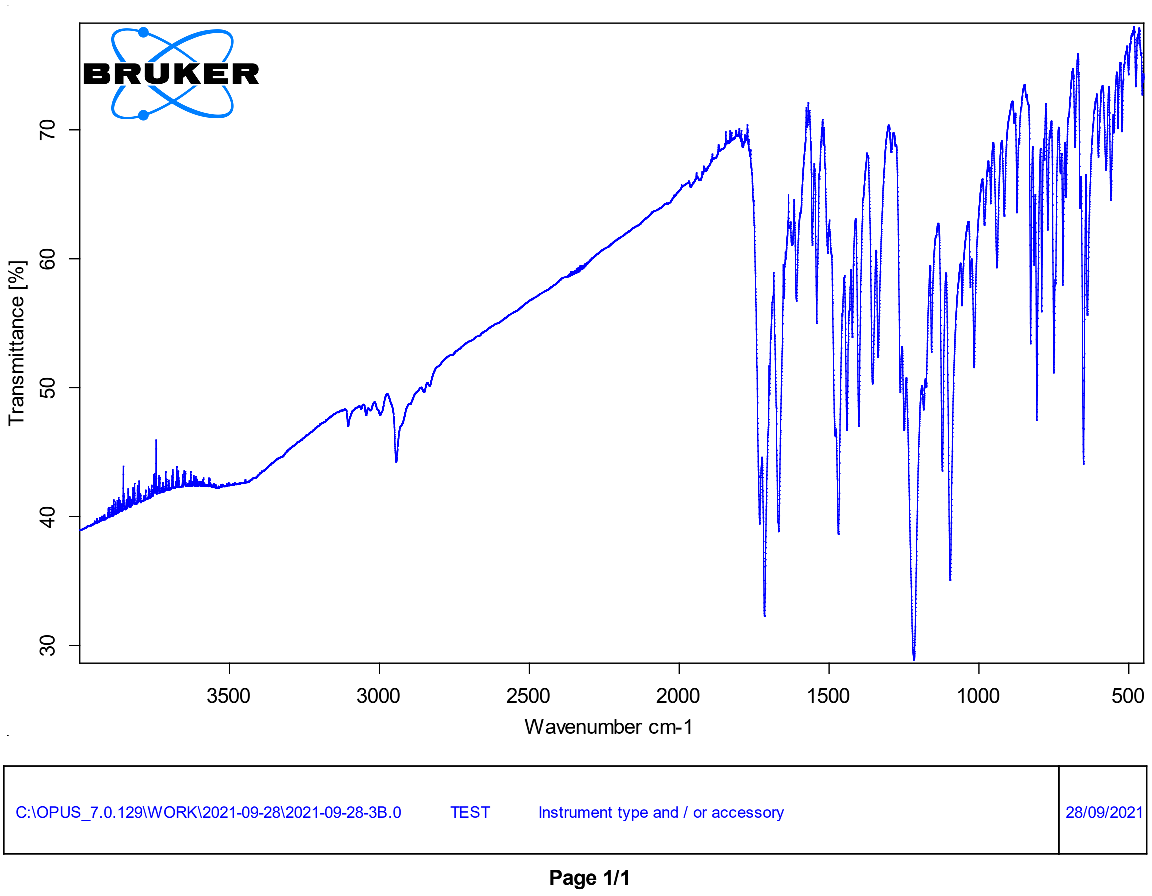

Figure S17. IR spectrum of the compound **3b.**

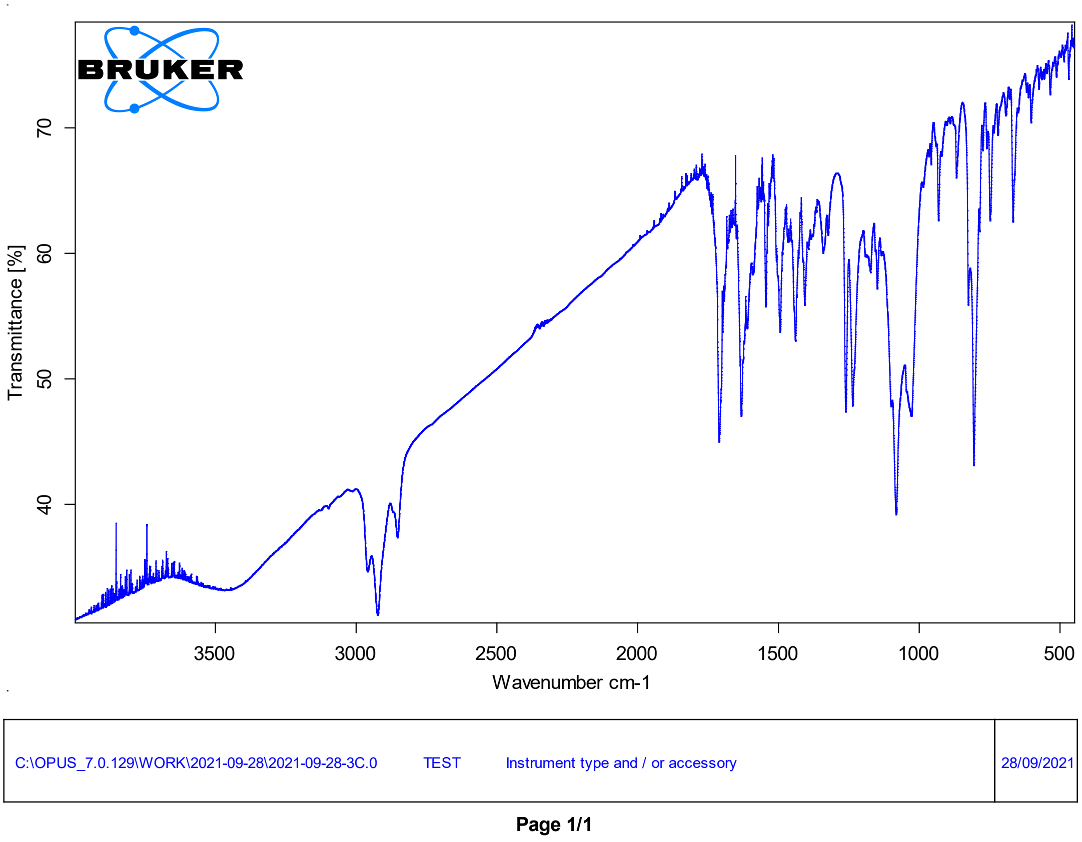

Figure S18. IR spectrum of the compound **3c.**

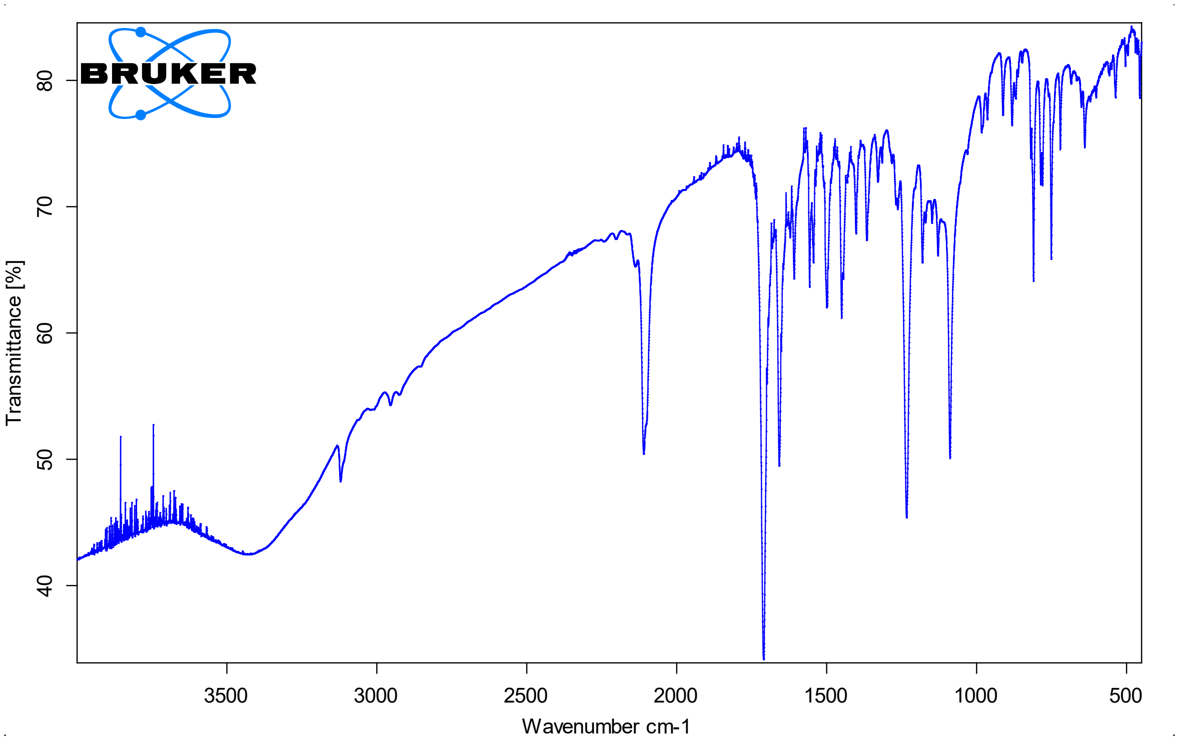

C:\OPUS_7.0.129\WORK\2021-09-28\2021-09-28-4A.0 TEST Instrument type and / or accessory 28/09/2021

Page 1/1

Figure S19. IR spectrum of the compound **4a.**

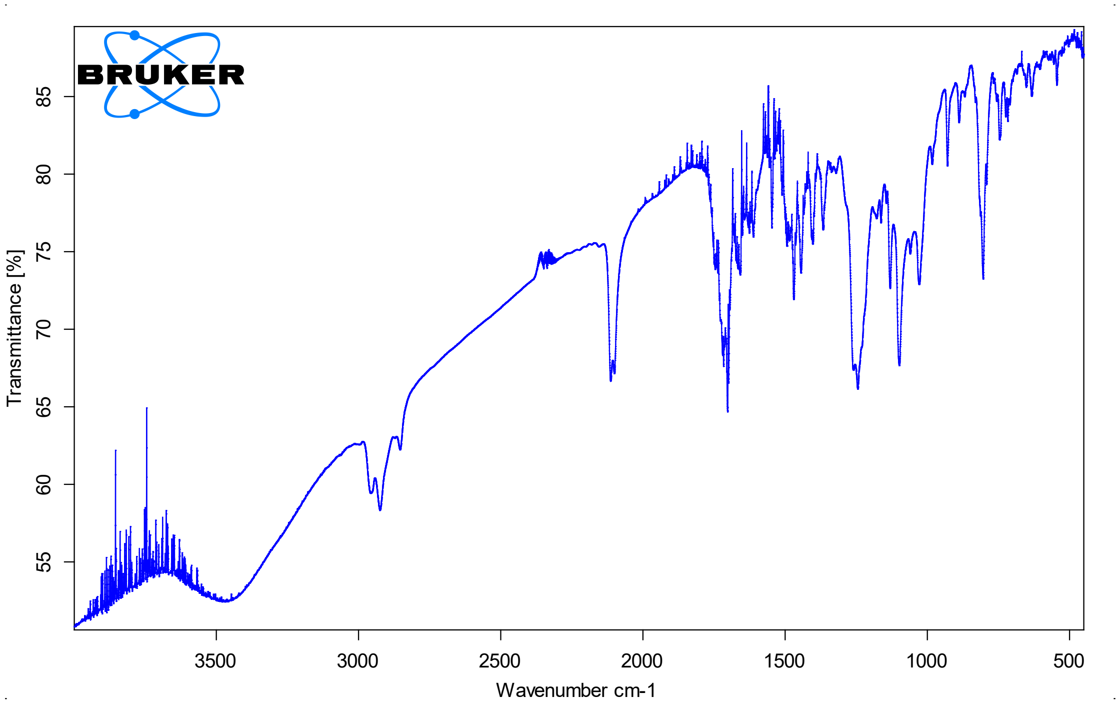

C:\OPUS_7.0.129\WORK\2021-09-28\2021-09-28-4B.0 TEST Instrument type and / or accessory 28/09/2021

Page 1/1

Figure S20. IR spectrum of the compound **4b.**

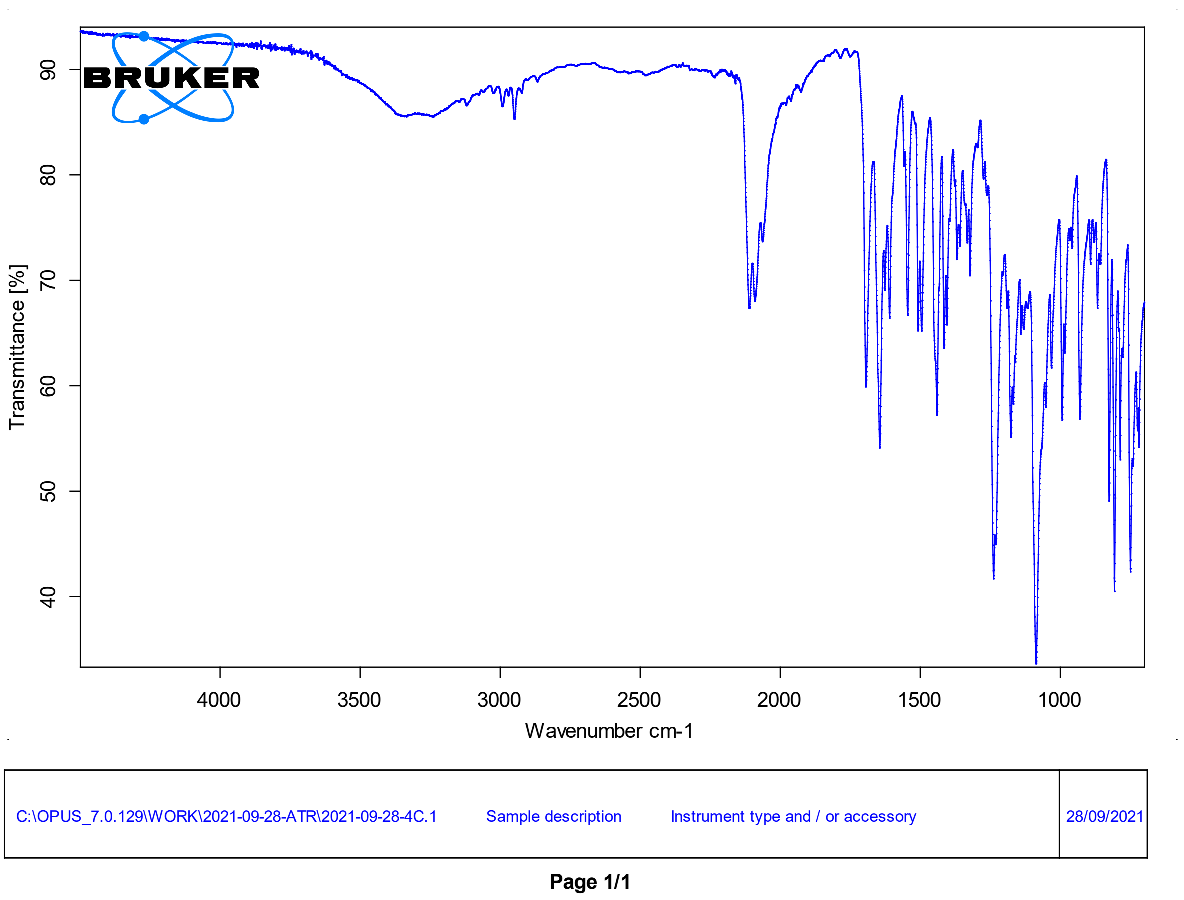

Figure S21. IR spectrum of the compound **4c.**

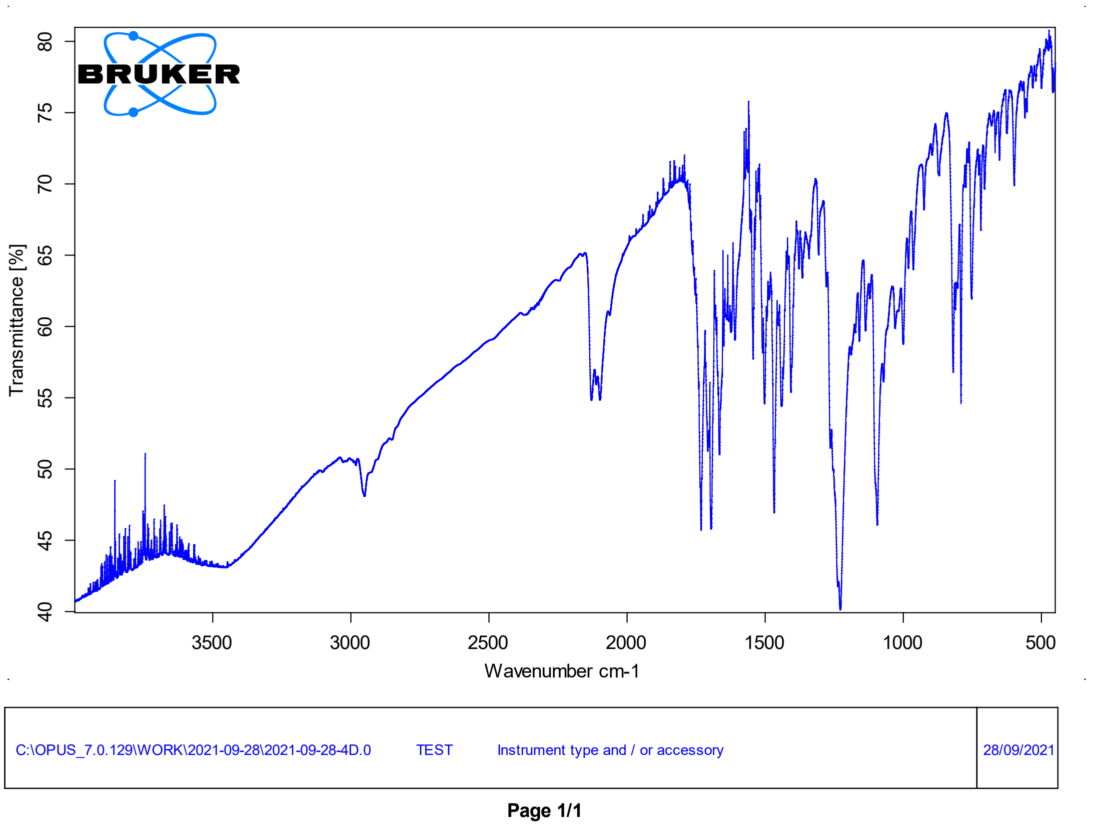

Figure S22. IR spectrum of the compound **4d.**

4. UV spectra of the obtained compounds

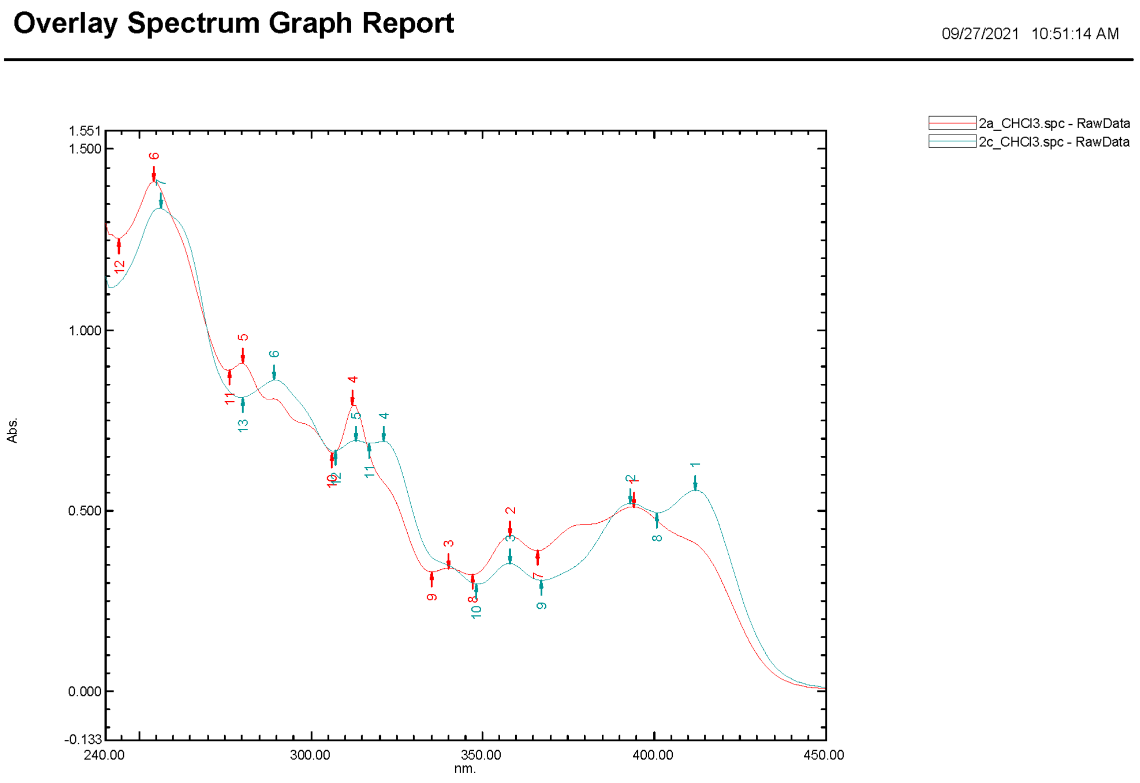

Figure S23. Overlayed UV-Vis spectra of the compounds **2a** and **2c** in chloroform.

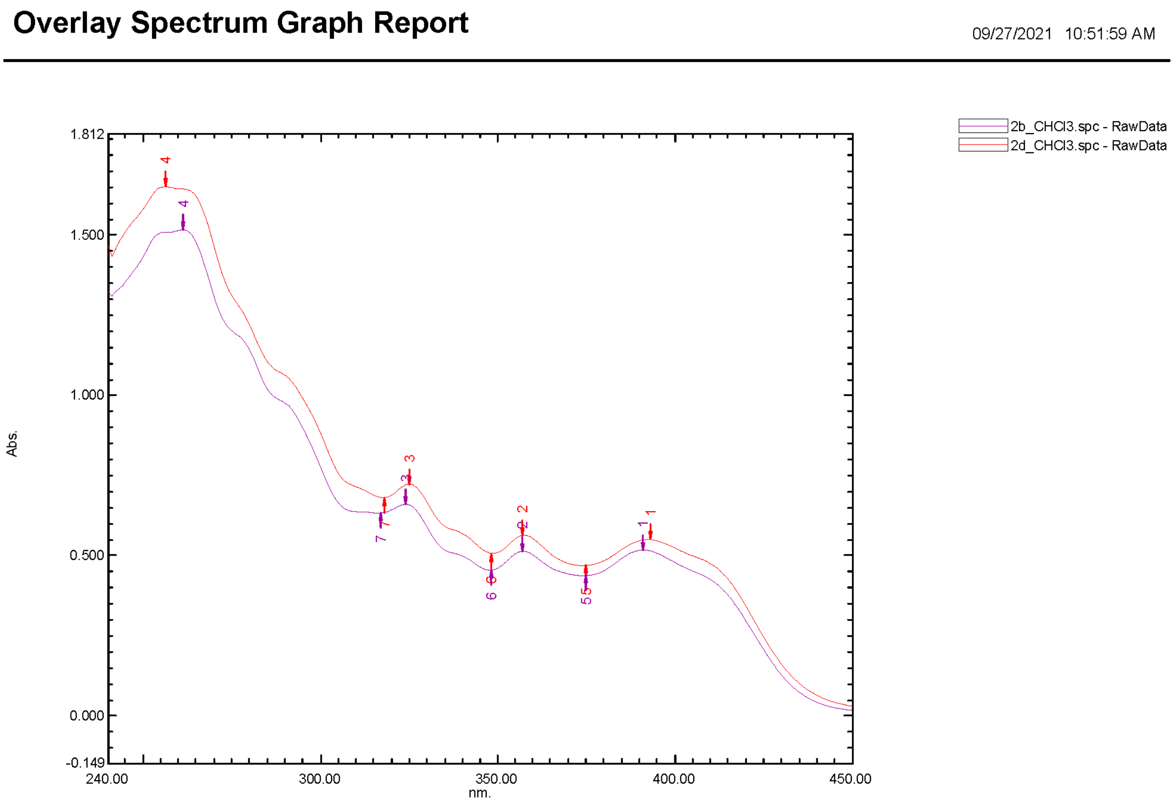

Figure S24. Overlayed UV-Vis spectra of the compounds **2b** and **2d** in chloroform.

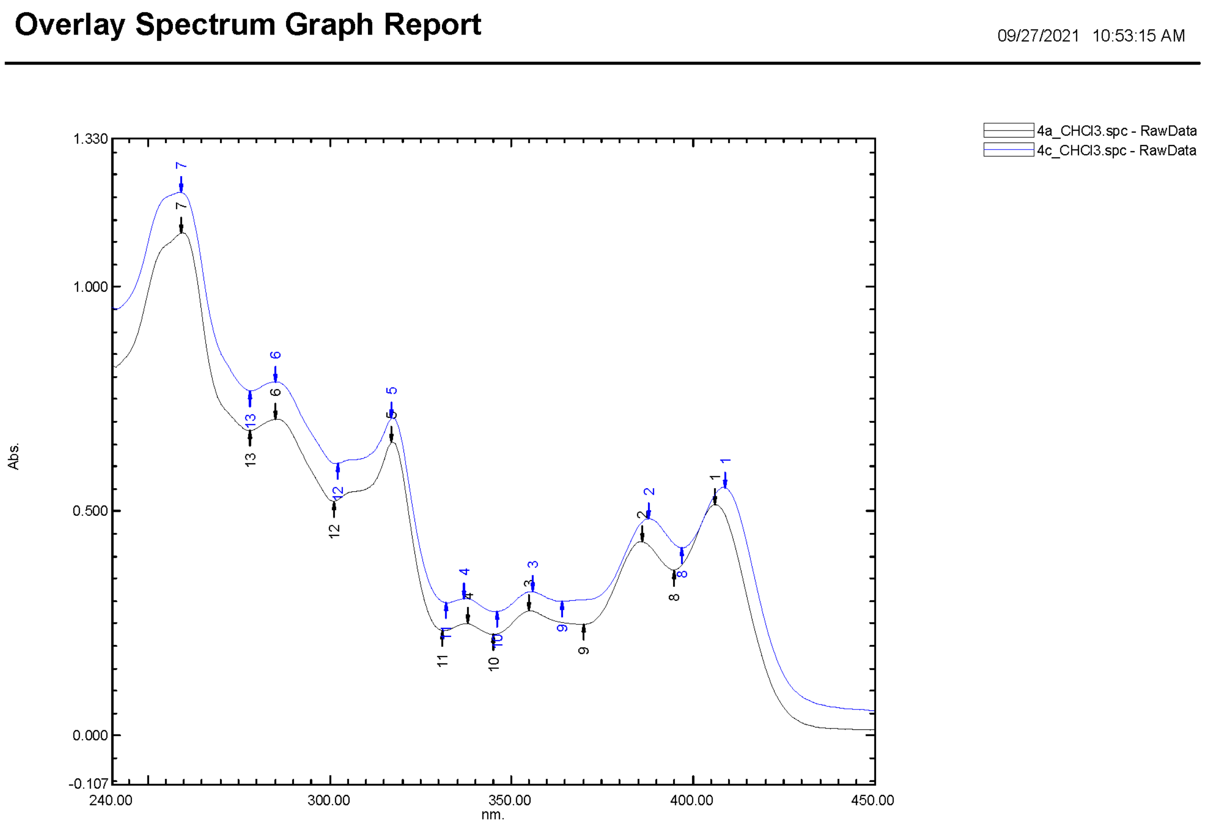

Figure S25. Overlayed UV-Vis spectra of the compounds **4a** and **4c** in chloroform.

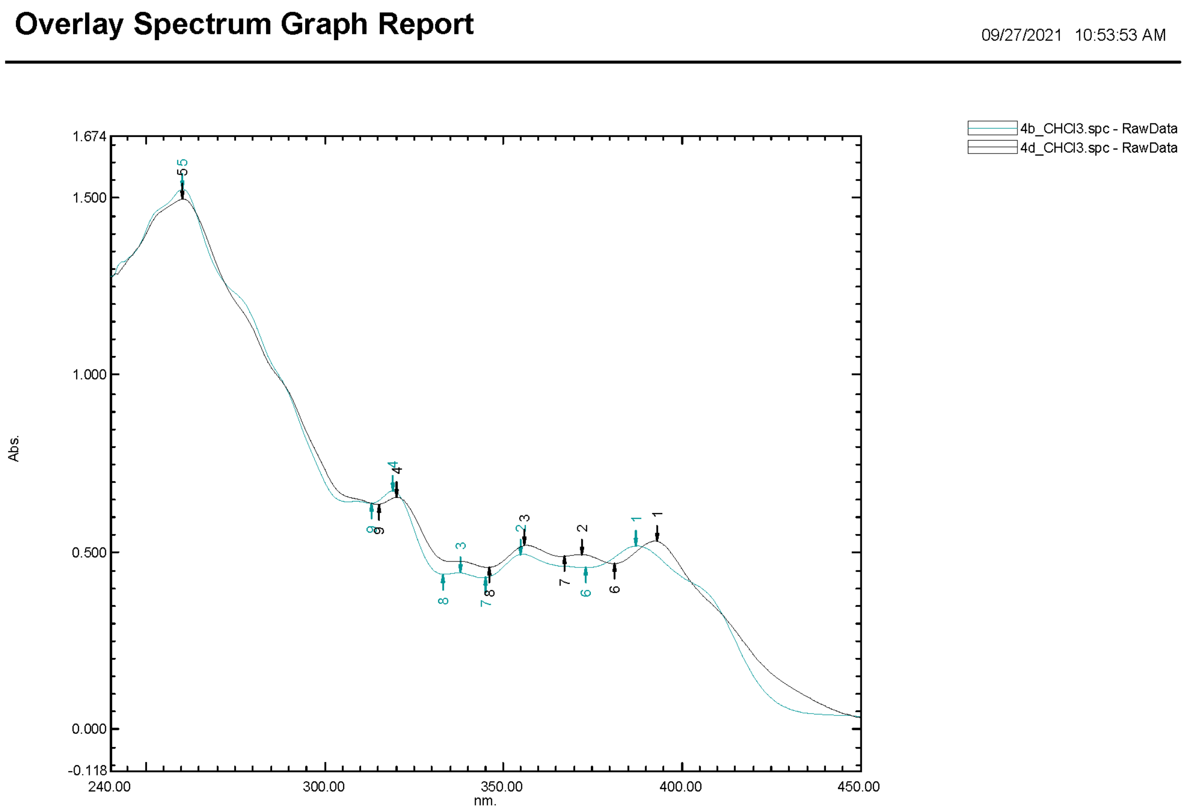

Figure S26. Overlayed UV-Vis spectra of the compounds **4b** and **4c** in chloroform.

In the case of the samples **2a-d** the profile of the third absorption region and the absorption maxima are similar for the samples **2a**, **2b** and **2d** (394 nm for **2a**, 391 nm for **2b** and 393 nm for **2d**), while for the sample **2c** the maximum of the absorption is situated on 412 nm. Sample **2c** present the same absorption bands like **2a**, **2b** and **2d** with a small bathochromic shift.

In the case of the samples **4a** and **4c** the profile of the third absorption region and the absorption maxima are similar (406 nm for **4a** and 409 nm for **4c**), while the samples **4b** and **4d** have similar profile of the third absorption region and the absorption maxima (387 nm for **4b** and 393 nm for **4d**). Samples **4a** and **4c** present the same absorption bands like **4b** and **4d** with a small bathochromic shift. The similar absorption behaviour of the samples **4a** with **4c** and of the samples **4b** with **4d** can be explained by their similar structure (samples **b** and **d** having a supplementary carbomethoxy group in the 2nd position of the azatetracyclic skeleton in comparison with the samples **a** and **c**).

5. Absorption vs emission spectra of the obtained compounds

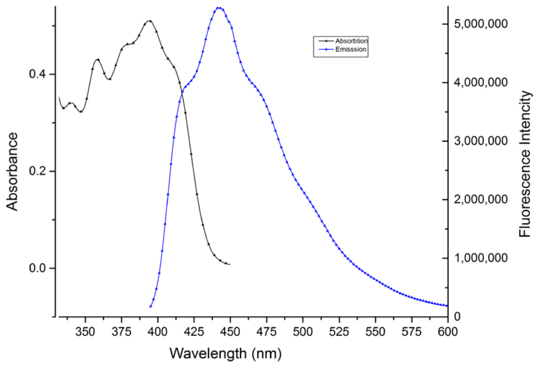

2a

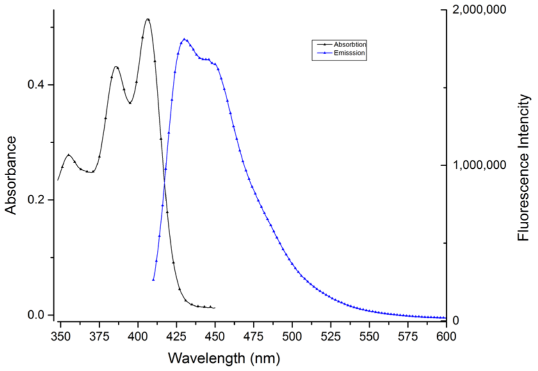

4a

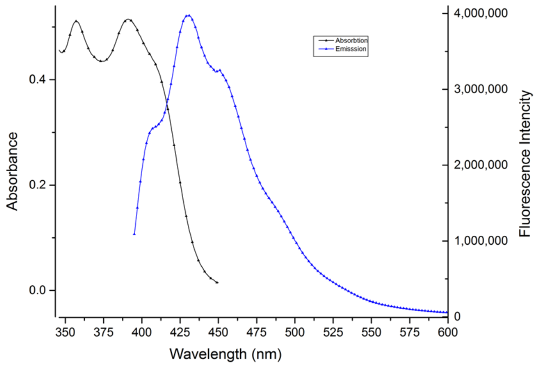

2b

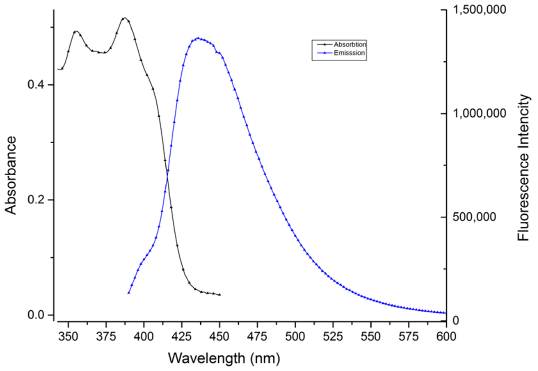

4b

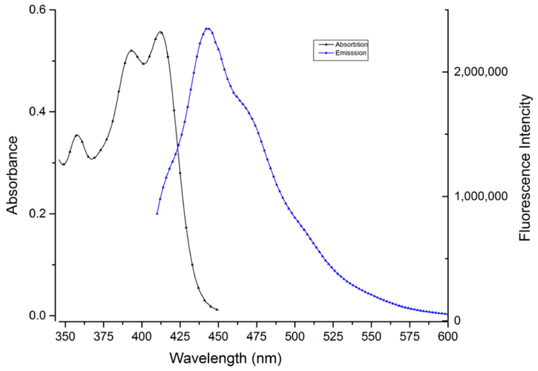

2c

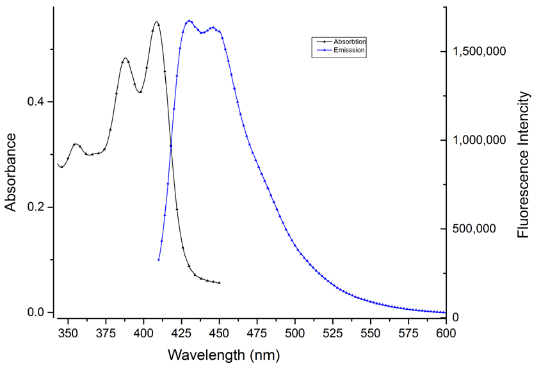

4c

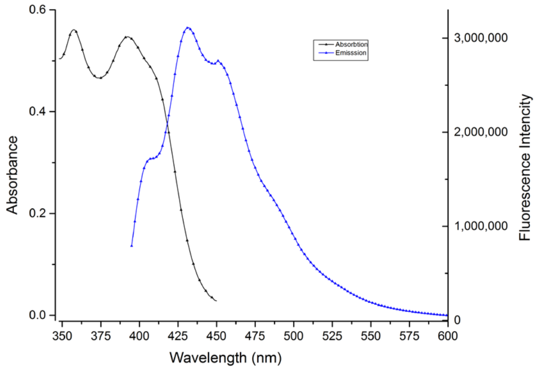

2d

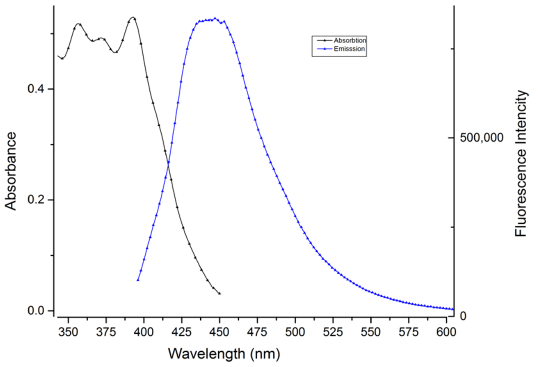

4d

Figure S27. The absorption (black line) and emission (blue line) spectra of functionalized derivatives: bromides **2a-d** (left column) and azides **4a-d** (right column). Excitation wavelength was: 380 nm for sample **4b**; 385 nm for samples **2a**, **2b**, **2d** and **4d**; and 400 nm for samples **2c**, **4a** and **4c**.

In the case of the bromides **2a-d**, the samples **2a** and **2c** have similar peak profile with λ_{max} = 443 nm for sample **2a** and respectively λ_{max} = 444 nm for sample **2c**. The fluorescence intensity of the sample **2a** is higher than the fluorescence intensity of the sample **2c**. The samples **2b** and **2d** have similar peak profile having λ_{max} = 431 nm for sample **2b** and respectively λ_{max} = 433 nm for sample **2d**. The fluorescence intensity of the sample **2b** is higher than the fluorescence intensity of the sample **2d**, but lower than the fluorescence intensity of the sample **2c**. The fluorescence intensity of all bromides **2a-d** is higher than the fluorescence of the corresponding azides **4a-d**.

In the case of the azides **4a-d**, the samples **4a** and **4c** have structured band with similar peak profile (λ_{max} = 430 nm for both sample **4a** and **4c**) and intensity. The azides **4b** and **4d** have similar peak profile (λ_{max} = 435 nm for sample **4b** and respectively λ_{max} = 445 nm for sample **4d**) with unstructured band. The fluorescence intensity of the sample **4d** is lower (almost a half) than the intensity of the sample **4b**. Sample **4a** have the highest fluorescence intensity from all the azides.
